# Geroprotective Effects of Drugs Modulating Metabolic Pathways: Perspectives of Pharmacology in Anti-Aging Therapy

**DOI:** 10.3390/ijms27177521

**Published:** 2026-08-22

**Authors:** Marta Grycan, Rafał Zyśk, Gabriela Grycan, Grzegorz Jakiel, Alicja Dudek, Grażyna Gromadzka

**Affiliations:** 1 Faculty of Medicine, Maria Skłodowska-Curie Medical University, Al. Solidarności 12, 03-411 Warsaw, Poland; 2 Department of Pharmacology and Clinical Pharmacology, Faculty of Medicine, Collegium Medicum, Cardinal Stefan Wyszynski University in Warsaw, Wóycickiego 1/3, 01-938 Warsaw, Poland; 3 1st Department of Obstetrics and Gynecology, Medical Centre of Postgraduate Medical Education, Żelazna 90, 01-004 Warsaw, Poland; 4 Department of Endocrinology, Medical Centre of Postgraduate Medical Education, Bielanski Hospital, Cegłowska 80, 01-809 Warsaw, Poland; 5 Department of Biomedical Sciences, Faculty of Medicine, Collegium Medicum, Cardinal Stefan Wyszynski University in Warsaw, Wóycickiego Street 1/3, 01-938 Warsaw, Poland

**Keywords:** longevity, metformin, rapamycin, SGLT2 inhibitors, GLP-1 receptor agonists, statins, senolytics, taurine, NAD^+^ precursors, menopausal hormone therapy, geroprotection, geroprotectors

## Abstract

Aging is the strongest risk factor for chronic diseases such as cardiovascular diseases, cancer, diabetes, and neurodegenerative disorders. Advances in geroscience indicate that pharmacological modulation of conserved molecular pathways may extend healthspan and delay multimorbidity. A structured narrative review of the PubMed, Scopus, and Web of Science literature published between January 2010 and May 2026 was conducted, with seminal earlier studies retained where relevant. The review focused on molecular pathways implicated in aging, pharmacological interventions targeting these pathways, and their preclinical and clinical evaluation. Particular emphasis was placed on translational evidence, including human biomarker studies and randomized clinical trials, and on the distinction between biomarker modulation and clinically meaningful outcomes. Repurposed drugs such as metformin and rapamycin have among the most extensive preclinical and translational evidence, although clinical evidence for broadly applicable geroprotection remains limited. Statins, SGLT2 inhibitors, GLP-1 receptor agonists, and menopausal hormone therapy have established disease-specific or cardiometabolic benefits that may have indirect relevance to geroprotection, but direct effects on biological aging and healthspan remain unproven. Other candidates, including senolytics, NAD^+^ precursors, taurine, and epigenetic reprogramming approaches, are at different stages of translational development, with evidence ranging from promising preclinical findings to early human studies. Across interventions, a substantial gap remains between mechanistic plausibility and clinically validated geroprotection. Geroprotective pharmacology represents a promising but incompletely validated approach to extending healthspan. Major uncertainties include the absence of universally accepted biomarkers and clinical endpoints of biological aging, heterogeneity in treatment response, optimal timing and duration of interventions, and long-term safety. Future research should prioritize adequately powered randomized clinical trials integrating standardized measures of biological aging with clinically meaningful outcomes, alongside biomarker-guided patient selection, appropriate treatment timing, and careful assessment of long-term safety. The future of geroprotective medicine will depend not only on identifying additional pharmacological targets, but on demonstrating that their modulation produces durable and clinically meaningful benefits in humans.

## 1. Introduction

Aging is the strongest risk factor for the development of major chronic diseases, including cardiovascular diseases, cancer, diabetes, and neurodegenerative disorders [1]. For decades, aging was considered an inevitable biological process that could not be modified. However, advances in molecular biology and geroscience have fundamentally changed this paradigm. Aging is currently recognized as a multifactorial and potentially modifiable biological process influenced by genetic, metabolic, environmental, and behavioral factors [2,3]. Significant progress has also been made in developing methods to measure and monitor biological aging. Biomarkers of biological age now enable a more precise assessment of geroprotective interventions and may facilitate future translational research [4,5,6].

The rapid development of geroscience is gradually shifting the medical perspective on aging—from viewing it solely as a risk factor to recognizing it as a potential therapeutic target. This concept was initially proposed by Vladimir M. Dilman in 1971 and was subsequently developed by other researchers [7,8,9]. An important conceptual framework that integrated previously described mechanisms of aging into a unified conceptual model was the “Hallmarks of Aging” framework. Although this framework remains one of the most frequently used references in geroscience, it should primarily be regarded as a conceptual synthesis rather than a discovery of novel biological mechanisms [1].

At the same time, the longevity field has expanded rapidly, often accompanied by substantial commercial interest and highly publicized claims that have not always been supported by reproducible evidence [10]. Several initially promising findings in aging research have later proven difficult to reproduce independently or have demonstrated substantially smaller effect sizes than initially reported [11,12]. Consequently, rigorous methodology, independent validation, and cautious interpretation of early findings remain essential for translating geroscience into evidence-based clinical practice [13].

The key molecular pathways involved in aging include mechanistic Target of Rapamycin (mTOR), AMP-activated protein kinase (AMPK), insulin/IGF-1 signaling, nicotinamide adenine dinucleotide (NAD^+^) metabolism, and molecular pathways associated with cellular senescence [14,15,16]. Experimental evidence indicates that pharmacological modulation of these pathways may extend lifespan and healthspan across multiple experimental models, ranging from yeast and nematodes to rodents and non-human primates [17,18].

Several drugs already clinically approved, originally developed for other indications, have been identified as promising geroprotective candidates. Metformin, an AMPK activator widely used in the treatment of type 2 diabetes, has been associated in observational studies with a reduction in the risk of cancer, cardiovascular diseases, and overall mortality [19,20]. The planned TAME (Targeting Aging with Metformin) trial is designed to evaluate whether metformin can delay the onset of age-related diseases in older adults without diabetes [21]. Another example is rapamycin—an mTOR inhibitor that consistently extended lifespan in mouse studies [22]. In clinical studies, low doses of rapamycin or its analogs demonstrated beneficial immunomodulatory effects and the potential to limit aging-associated secretory phenotypes, Senescence-Associated Secretory Phenotype (SASP), but long-term safety remains a challenge [23,24].

In parallel, the discovery of senolytics—natural or synthetic substances selectively eliminating senescent cells, sometimes referred to as “zombie” cells—opened a new direction of research. It is precisely senescent cells, which have permanently arrested the cell cycle and activated numerous Senescent Cell Anti-Apoptotic Pathways (SCAPs), that play a key role in the aging process. In particular, SASP-positive senescent cells are responsible for inflammaging as well as degradation of the extracellular matrix and collagen fibers [25,26,27].

Preclinical studies have demonstrated that senolytic interventions may improve physical performance, delay multiple age-related pathologies, and extend lifespan [28,29]. Early pilot studies in humans with chronic diseases suggest that senolytics may reduce the values of aging biomarkers, although robust clinical evidence remains limited [30].

Despite these advances, the translation of geroprotective pharmacology into clinical practice faces significant barriers: the lack of scientific consensus regarding universal biomarkers of aging, the need for long-term and costly randomized studies, and ethical issues related to the medicalization of aging [31]. An additional problem is the lack of interest from the pharmaceutical industry in clinical trials conducted to evaluate the effectiveness of naturally derived substances, for which the possibilities of patent protection are limited.

In this review, we critically summarize current knowledge regarding pharmacological strategies targeting aging, with particular emphasis on molecular mechanisms, preclinical and clinical evidence, translational challenges, and future directions in geroprotective pharmacology.

## 2. Materials and Methods

This review was prepared using a structured narrative approach with elements of systematic literature selection to provide a comprehensive and critical overview of pharmacological interventions targeting molecular pathways involved in aging and longevity regulation. The review was developed in accordance with the recommendations of the Scale for the Assessment of Narrative Review Articles (SANRA), which guided its overall structure, critical appraisal of the literature, and presentation of evidence.

Rather than attempting a quantitative synthesis of the available evidence, the objective was to integrate mechanistic, experimental, preclinical, and clinical findings concerning pharmacological geroprotection, while distinguishing established clinical evidence from mechanistic plausibility and early-stage translational findings. Particular attention was given to the strength and maturity of evidence supporting individual candidate geroprotective interventions, as well as to areas of uncertainty and current limitations of clinical translation.

### 2.1. Literature Search Strategy

A comprehensive literature search was performed using the PubMed/MEDLINE, Scopus, and Web of Science databases. Publications published between January 2010 and March 2026 were considered, while seminal earlier studies were retained when necessary to provide essential mechanistic or historical context. Literature screening and selection were performed between 1 November 2025 and 1 March 2026. Clinical trial registry information was additionally checked against available registry records during manuscript preparation to update the status of ongoing and recently completed studies.

The search strategy incorporated combinations of Medical Subject Headings (MeSH) and free-text keywords, including “aging”, “geroprotection”, “geroprotectors”, “longevity”, “healthspan”, “metformin”, “rapamycin”, “mTOR”, “AMPK”, “cellular senescence”, “senolytics”, “SASP”, “NAD^+^”, “nicotinamide riboside”, “nicotinamide mononucleotide”, “taurine”, “SGLT2 inhibitors”, “GLP-1 receptor agonists”, “mitochondrial dysfunction”, “inflammaging”, and “anti-aging therapy”. Boolean operators (AND, OR) were applied to optimize search specificity and sensitivity.

The search was designed to identify literature spanning the biological mechanisms of aging, pharmacological interventions targeting these mechanisms, and their preclinical and clinical evaluation. Retrieved records were screened for relevance to the predefined scope of the review, with particular attention to studies providing mechanistic, experimental, biomarker, observational, or randomized clinical evidence. Because this was a structured narrative review rather than a systematic review, the search was iterative and reference selection was guided by scientific relevance, methodological quality, recency, and contribution to the translational assessment of candidate geroprotective interventions.

Additional relevant publications were identified through reference lists of selected systematic reviews, meta-analyses, and key original studies when they provided important mechanistic, clinical, or historical context.

The final reference set was therefore not intended to represent an exhaustive enumeration of all publications identified by the searches, but a focused selection of studies considered most informative for the mechanistic and translational objectives of the review.

### 2.2. Eligibility Criteria

The review included:

∗ original experimental and preclinical studies;
∗ observational studies involving human populations;
∗ randomized and non-randomized clinical trials;
∗ systematic reviews and meta-analyses;
∗ selected high-quality narrative reviews relevant to geroscience and pharmacological geroprotection.

Priority was given to studies published within the last 5–10 years, while seminal earlier publications were retained when essential for understanding fundamental molecular mechanisms or the historical development of specific geroprotective concepts.

Studies were selected according to their relevance to the predefined scope of the review, methodological quality, and contribution to the current understanding of pharmacological modulation of aging-related pathways. Particular emphasis was placed on studies investigating conserved molecular pathways involved in aging regulation, pharmacological modulation of cellular senescence and SASP, biomarkers of biological aging, and interventions with potential geroprotective activity.

When multiple publications addressed the same intervention or biological mechanism, priority was given to randomized clinical trials, larger human studies, systematic reviews and meta-analyses, and studies providing clinically meaningful or mechanistically informative outcomes.

Non-English publications, conference abstracts without available full-text data, duplicate publications, and studies lacking clear mechanistic, pharmacological, or translational relevance to aging and geroprotection were excluded.

### 2.3. Data Extraction and Critical Synthesis

Selected publications were evaluated with particular attention to molecular pathways implicated in aging and longevity regulation, including mTOR, AMPK, sirtuins/NAD^+^, NF-κB, cellular senescence, autophagy–lysosomal pathways, and mitochondrial regulation;

- pharmacological mechanisms of candidate geroprotective interventions;
- evidence derived from cellular and animal models;
- observational evidence in human populations;
- biomarker-based human studies;
- randomized and non-randomized clinical trials;
- clinically meaningful outcomes, including multimorbidity, functional decline, cardio-vascular and metabolic outcomes, mortality, and healthspan-related measures;
- safety, tolerability, and limitations relevant to long-term preventive use.

Rather than providing only a descriptive summary of individual studies, the evidence was synthesized according to the strength, consistency, and maturity of the available data. Particular emphasis was placed on distinguishing mechanistic plausibility and preclinical efficacy from biomarker modulation in humans and from clinically demonstrated therapeutic benefit.

Interventions supported predominantly by cellular or animal studies were therefore interpreted separately from those for which observational human data, biomarker studies, or randomized clinical evidence were available. This approach was intended to avoid equating modulation of aging-related pathways with clinically validated geroprotective efficacy.

To facilitate comparison across interventions, the evidence was additionally organized according to preclinical findings, human observational evidence, clinical trial evidence, biomarker evidence, overall evidence strength, and major limitations, as summarized in Section 5.7. The evidence-strength classification represents a qualitative assessment by the authors based on the consistency, quality, and maturity of the available evidence and should not be interpreted as a formal grading system such as GRADE.

Because the manuscript was designed as a structured narrative review rather than a systematic review or meta-analysis, formal procedures such as PRISMA-based study selection, quantitative pooling of results, and formal risk-of-bias assessment were not applied.

## 3. Molecular and Cellular Pathways of Aging as Pharmacological Targets

Aging is driven by interconnected molecular processes disrupting cellular homeostasis. In recent years, several conserved pathways have been identified that play a central role in the regulation of longevity and healthy aging, while simultaneously constituting potential targets for pharmacology.

### 3.1. The mTOR Pathway

Mechanistic target of rapamycin (mTOR) integrates signals related to nutrient availability, cellular energy status, and growth factors, thereby regulating protein synthesis, metabolism, and autophagy. Among the two mTOR complexes, mTORC1 plays the predominant role in aging because it is closely associated with cellular senescence and the senescence-associated secretory phenotype (SASP) [32]. Chronic hyperactivation of the mTORC1 complex accelerates aging and promotes the development of age-related diseases, whereas its inhibition by rapamycin or rapalogs (e.g., everolimus and temsirolimus) extends lifespan in various animal species [32,33,34,35].

In mice, rapamycin extended median and maximal lifespan even when administered at an advanced age, which highlights its strong geroprotective potential [7,16]. However, chronic mTOR inhibition may adversely affect immune function and lipid metabolism, raising concerns regarding its long-term safety in humans [17].

From a translational perspective, mTOR currently represents one of the most strongly supported pharmacological targets in geroscience at the preclinical level, with growing but still incomplete human evidence. Rapamycin and rapalogs have produced reproducible lifespan and healthspan benefits in multiple experimental models, whereas clinical studies in humans have primarily evaluated safety, tolerability, or disease-specific outcomes rather than validated measures of delayed aging or extended healthspan [17]. Thus, mTOR inhibition represents a leading gerotherapeutic strategy, but its status as a clinically validated geroprotective intervention remains to be established.

### 3.2. AMPK Activation

AMP-activated protein kinase (AMPK) acts as a cellular energy sensor, activated when the AMP/ATP ratio increases. Once activated, AMPK promotes catabolic processes, including autophagy and fatty acid oxidation, while suppressing anabolic pathways such as lipid and protein synthesis, thereby counteracting age-related metabolic dysfunction [15,36,37]. In addition, AMPK inhibits mTOR and activates SIRT1. With aging, a decline in AMPK activity is observed, leading to lipid accumulation and decreased autophagy. Pharmacological activation of AMPK by metformin improves insulin sensitivity, reduces oxidative stress, and suppresses inflammation [38]. Additional AMPK-activating interventions include berberine, resveratrol, epigallocatechin gallate (EGCG), intermittent fasting, and regular physical activity.

Translationally, AMPK is supported by substantial human evidence because several clinically established interventions, most notably metformin, modulate this pathway. However, the available clinical evidence does not establish that AMPK activation itself produces a generalized geroprotective effect in humans. The pathway should therefore be considered clinically relevant and pharmacologically tractable, but the geroprotective consequences of its modulation remain incompletely validated.

### 3.3. Sirtuins and NAD^+^ Metabolism

Sirtuins (SIRT1-7) are a family of NAD^+^-dependent deacetylases regulating stress resistance, inflammation, mitochondrial function, DNA repair, and genomic stability. With aging, NAD^+^ levels decline, which decreases sirtuin activity, contributing to impaired DNA repair and metabolic disturbances [16,39,40]. Supplementation with NAD^+^ precursors such as nicotinamide riboside (NR) or nicotinamide mononucleotide (NMN) restores NAD^+^ reserves and improves healthy aging in animal models [41,42]. From the perspective of geroprotection, SIRT1, SIRT3, and SIRT6 appear to play a key role. The ability to activate sirtuins was demonstrated in experimental studies by: rapamycin (SIRT1, SIRT4), resveratrol (SIRT1-7), quercetin, fisetin (SIRT1, SIRT6), curcumin (SIRT1), EGCG (SIRT1), and salidroside (SIRT3) [35]. However, despite encouraging findings in experimental models and selected clinical studies, natural sirtuin activators are characterized by poor bioavailability and have not demonstrated consistent clinically meaningful geroprotective effects in humans. The first clinically developed synthetic SIRT6 activator is forvisirvat, but clinical studies evaluating this drug are focused on its use in the treatment of adults with major depressive disorder [43]. At present, there are no data regarding the potential geroprotective activity of forvisirvat.

The history of sirtuin activators illustrates that promising mechanistic discoveries do not necessarily translate into clinically meaningful geroprotective interventions. The development of synthetic and natural sirtuin activators also illustrates the limitations of translating promising molecular targets into clinically effective geroprotective therapies, emphasizing the need for independent replication before broad clinical implementation.

Despite strong mechanistic and preclinical rationale, the translational evidence supporting sirtuin activation or NAD^+^ restoration as geroprotective strategies remains limited. Human studies of NAD^+^ precursors and related interventions have generally demonstrated changes in selected metabolic or molecular biomarkers, but evidence that these effects translate into delayed multimorbidity, improved functional aging, or extended healthspan remains insufficient. Accordingly, sirtuins and NAD^+^ metabolism should currently be regarded as promising but predominantly investigational geroscience targets.

### 3.4. The NF-κB (Nuclear Factor Kappa-B) Pathway

NF-κB is a family of transcription factors that integrates signals from TNF, IL-1, TLRs, RAGE, and antigen receptors. Numerous studies have demonstrated that NF-κB signaling is a central regulator of chronic inflammation and plays a pivotal role in age-associated inflammaging. With aging, persistent activation of this pathway promotes inflammatory signaling, influences the fate of tissue cells toward senescence, and may contribute to the maintenance of a chronic pro-inflammatory environment [44]. NF-κB can be activated by reactive oxygen species (ROS), TNF-α, and factors associated with the senescence-associated secretory phenotype (SASP). Experimental studies suggest that compounds such as metformin, curcumin, resveratrol, berberine, epigallocatechin gallate (EGCG), and omega-3 fatty acids may attenuate NF-κB signaling, thereby reducing inflammatory and oxidative responses [45].

From a translational perspective, however, NF-κB should currently be regarded as a biologically compelling but clinically insufficiently validated geroprotective target. Its central role in inflammaging provides a strong mechanistic rationale for pharmacological modulation, but inhibition of NF-κB is not equivalent to demonstrated modification of the overall aging process. Most available evidence concerns modulation of inflammatory signaling or disease-specific outcomes rather than validated measures of biological aging, healthspan, or multimorbidity. This distinction is particularly important because NF-κB participates in diverse physiological processes, including immune responses and tissue homeostasis, making sustained systemic inhibition potentially problematic.

The clinical relevance of NF-κB modulation is therefore currently best considered in the context of interventions acting through multiple interconnected pathways. Metformin, for example, has been reported to inhibit NF-κB signaling and reduce pro-inflammatory cytokine production, while its broader effects on AMPK, mitochondrial function, and mTOR signaling may contribute to its potential geroprotective activity [46]. Nevertheless, the absence of definitive evidence that selective NF-κB modulation prolongs human healthspan or delays multimorbidity indicates that NF-κB remains primarily a mechanistic target rather than a clinically validated geroprotective pathway. This illustrates a broader principle of geroscience: strong biological plausibility and modulation of an aging-associated pathway do not necessarily translate into clinically meaningful geroprotection.

### 3.5. The p53/p21/p16INK4a Pathway (Cell Cycle Control and Cellular Senescence)

Activation of the p53/p21/p16INK4a pathway represents a cellular response to oxidative stress and unrepaired DNA damage, leading to irreversible cell-cycle arrest (cellular senescence) and activation of senescent cell anti-apoptotic pathways (SCAPs). In a cell in which apoptosis has not occurred despite unrepaired damage, this mechanism prevents carcinogenesis. Senescent cells accumulate in most human tissues with age. Some of them secrete SASP—a set of pro-inflammatory and collagen fiber-degrading factors (including pro-inflammatory cytokines, chemokines, proteases, growth factors, and proliferation modulators), which contributes to tissue dysfunction [47].

For some SASP proteins, a strong association with multiple features of aging in humans has been demonstrated, making them valuable biomarkers of aging. These include: insulin-like growth factor binding protein 2 (IGFBP2), growth differentiation factor 15 (GDF15), cystatin-C, C1 esterase inhibitor, 14.3.3 protein, TIMP metallopeptidase inhibitor 1, and cathepsin Z [48]. Genetic or pharmacological, selective elimination of senescent cells through deactivation of SCAPs delays the development of many age-related diseases and improves healthy aging in animals [28,49,50]. This led to the discovery of senolytics (e.g., dasatinib, quercetin, fisetin, and navitoclax) and senomorphics (e.g., metformin and EGCG), that is, substances suppressing SASP activity without eliminating senescent cells, as new pharmacological classes [51].

Among the emerging geroscience targets, cellular senescence and the SASP have generated substantial translational interest because senescent-cell accumulation and inflammatory signaling can be directly targeted pharmacologically. Nevertheless, the clinical evidence remains early and heterogeneous, with available studies primarily focusing on safety, feasibility, biomarkers, or disease-specific outcomes. Senolytic and senomorphic strategies therefore represent promising translational candidates, but their ability to produce durable improvements in human healthspan has not yet been established.

### 3.6. The ALP (Autophagy–Lysosome Pathway)

The primary function of the autophagy–lysosome pathway (ALP) is the removal and recycling of damaged organelles and protein aggregates, thereby maintaining cellular proteostasis. With aging, a progressive decline in autophagy activity is observed, leading to the accumulation of damaged structures (e.g., accumulation of lipofuscin), followed by disruption of proteostasis and chronic cellular stress.

Interventions that activate ALP include rapamycin, spermidine, metformin, trehalose, resveratrol, berberine, EGCG, urolithin A, intermittent fasting, and physical activity. One study conducted in an animal model demonstrated that clomiphene citrate activated ALP via transcription factor EB (TFEB) [52].

The translational relevance of autophagy modulation is biologically compelling but remains difficult to establish clinically. Although enhancement of autophagic and lysosomal function is consistently associated with healthy aging in experimental models, clinical studies have rarely demonstrated that pharmacological activation of autophagy directly improves validated measures of biological aging or healthspan. Autophagy should therefore be considered an important mechanistic component of geroprotection, while its status as an independent clinical therapeutic target remains uncertain.

### 3.7. The PGC-1α (Peroxisome Proliferator-Activated Receptor Gamma Coactivator 1-Alpha) Pathway

PGC-1α is a transcriptional coactivator involved in the regulation of cellular metabolism, primarily in mitochondrial function and mitochondrial biogenesis. PGC-1α stimulates the expression of genes responsible for the respiratory chain and regulates the response to energy signals. Consequently, cells become more efficient in ATP production and better adapted to metabolic stress. With aging, a decline in PGC-1α expression in human skeletal muscles is observed. Due to the fact that PGC-1αmodulates mitochondrial biogenesis (“mitochondrial quality control”) and participates in mitophagy, its activation is considered to limit mitochondrial dysfunction and may beneficially affect aging processes and age-related diseases such as sarcopenia, neurodegenerative diseases, and cardiovascular diseases [53]. In older individuals, decreased levels of PGC-1αoccur together with associated deterioration of mitochondrial function in muscles and reduced ATP levels, which may contribute to loss of muscle mass, strength, and performance. Studies show that physical activity may partially restore PGC-1α expression in muscle tissue [54]. Interventions that modulated PGC-1α in experimental models included metformin, resveratrol, curcumin, berberine, quercetin, melatonin, and taurine [53,55].

However, clinical validation of PGC-1α modulation as a pharmacological geroprotective strategy remains limited, and current evidence is largely derived from mechanistic, exercise-related, and preclinical studies.

### 3.8. Translational Relevance of Aging-Related Pathways

Although the pathways discussed above are highly interconnected and collectively provide a strong biological rationale for pharmacological geroprotection, their translational maturity is not equivalent. Among these targets, nutrient-sensing pathways such as mTOR and AMPK have progressed furthest toward human clinical evaluation, largely because they can be pharmacologically modulated using established drugs such as rapamycin and metformin. However, even for these relatively advanced targets, clinical evidence for generalized geroprotection remains incomplete. Sirtuin and NAD^+^-related pathways have strong mechanistic and preclinical support, but human studies have predominantly demonstrated effects on metabolic or molecular biomarkers rather than validated measures of delayed biological aging or healthspan. Similarly, autophagy and mitochondrial quality-control pathways have substantial experimental support but remain difficult to validate clinically because their modulation involves multiple interconnected processes and lacks standardized clinical endpoints [56].

NF-κB and inflammaging occupy a somewhat different position. Their biological relevance to age-related disease is supported by extensive evidence linking chronic inflammation with frailty, cardiovascular disease, neurodegeneration, and multimorbidity, but direct clinical validation of NF-κB inhibition as a generalized geroprotective strategy remains limited [25].

By contrast, pathways involved in cellular senescence, epigenetic remodeling, and partial cellular reprogramming remain particularly promising mechanistically but are further from clinical validation, owing in part to challenges related to target specificity, safety, and long-term consequences. This heterogeneity in translational maturity is important when interpreting candidate geroprotective interventions: a pathway may be strongly implicated in the biology of aging without yet representing a clinically validated therapeutic target.

The interconnected nature of these pathways also suggests that pharmacological effects are unlikely to be adequately captured by a single molecular or inflammatory biomarker. Interventions targeting one pathway may produce downstream effects across several hallmarks of aging, while the magnitude and direction of these effects may vary according to age, metabolic state, disease burden, and biological aging phenotype. Consequently, the translational value of a geroscience target should ultimately be judged not only by its mechanistic plausibility or ability to modify aging-associated biomarkers, but by reproducible evidence that such modulation improves clinically meaningful function, delays multimorbidity, or extends healthspan.

## 4. Drugs with Geroprotective Potential

Pharmacological interventions with geroprotective potential can be divided into two groups: repurposed drugs with an established safety profile already used for other indications, and novel candidates, drugs developed specifically in the context of geroprotection.

### 4.1. Drug Repurposing: From the Treatment of Chronic Diseases to Geroprotection

#### 4.1.1. Metformin

Metformin, the first-line pharmacological treatment for type 2 diabetes, is among the most extensively investigated candidate geroprotective agents [21,57]. Its mechanisms of action include AMPK activation, partial inhibition of mitochondrial complex I, and indirect suppression of mTOR signaling [35,58]. AMPK activation stimulates mitophagy and mitochondrial biogenesis through PGC-1α (peroxisome proliferator-activated receptor gamma coactivator 1-alpha) and ULK1 (Unc-51-like kinase 1), thereby supporting mitochondrial quality control. Importantly, modulation of evolutionarily conserved pathways such as AMPK does not necessarily translate into proportional clinical effects because aging results from simultaneous interactions among multiple biological systems.

Moreover, inhibition of mitochondrial complex I may reduce mitochondrial reactive oxygen species production and attenuate oxidative stress. Metformin also exerts anti-inflammatory effects through inhibition of NF-κB signaling and reduction in pro-inflammatory cytokine production [46]. Although metformin is not classified as a senolytic agent, several studies suggest that it may suppress SASP activity in senescent cells, indicating potential senomorphic properties.

Observational studies have reported lower incidence of cancer, cardiovascular diseases, and all-cause mortality among metformin users compared with diabetic patients treated with sulfonylureas and, in some analyses, compared with non-diabetic populations [19,20,59,60]. Metformin extended lifespan in several animal models and delayed selected age-related phenotypes [61]. However, definitive evidence demonstrating lifespan extension or prevention of multimorbidity in humans is currently lacking.

Kumazawa et al., in a study published in 2026, described a novel mechanism of metformin action occurring in senescent cells [62]. The researchers demonstrated in cellular and animal models that glucose limitation or metformin inhibits nuclear egress of cytoplasmic chromatin fragments (CCFs) formation. This study demonstrated that metformin in senescent cells also acts as a regulator of nuclear integrity, thereby indirectly inhibiting SASP and inflammaging.

Nevertheless, despite the growing mechanistic understanding of metformin action, translation into clinically meaningful slowing of biological aging remains uncertain [63]. Current evidence indicates that mechanistic plausibility alone cannot be considered sufficient evidence of clinical geroprotective efficacy [64], emphasizing the need for adequately powered randomized trials using validated biomarkers of biological aging [65].

Collectively, current evidence supports a transition from considering metformin a universal geroprotector toward viewing it as a potentially context-dependent intervention whose effectiveness may depend on age, metabolic phenotype, and biological aging status.

#### 4.1.2. Rapamycin and Rapalogs

Rapamycin, an mTOR inhibitor used as an immunosuppressive drug, belongs to the most potent lifespan-extending compounds in animal models [7,31]. Even when administered at an advanced age, it increases median and maximal lifespan in mice [22,66].

Gkioni et al. demonstrated in an animal model that the combination of rapamycin with the Ras–MEK–ERK pathway inhibitor trametinib acts even more strongly geroprotectively than rapamycin alone. The combination of both drugs improved median survival time of the animals by 34.9% in females and by 27.4% in males [67].

In 2026, Kell et al. provided evidence that rapamycin may exert direct genoprotective effects in the aging human immune system, beyond its canonical role as an mTOR inhibitor. In human immune cells, rapamycin reduced markers of DNA damage response and cellular senescence, suggesting enhanced resilience to genotoxic stress. These findings point toward a potential translational role for low-dose rapamycin in modulating immunosenescence in older adults [68]. In small clinical studies, low doses of rapamycin and everolimus improved selected aspects of immune function, particularly vaccine responses, without severe adverse effects [17]. Nevertheless, chronic inhibition of mTOR still raises certain concerns related to metabolic effects and immunosuppression [69].

On the other hand, some researchers suggest that rapamycin therapy at a dose of 0.5–1 mg daily or 5–7 mg weekly is sufficient to avoid adverse effects and reduce the risk of theoretical reconstitution of hyper-mTOR function [70].

Taken together, current human evidence supports the biological plausibility of rapamycin as a gerotherapeutic agent but remains insufficient to establish clinically meaningful slowing of aging or extension of human lifespan. Future randomized trials using validated biological aging biomarkers and clinically relevant outcomes are required before routine preventive use can be recommended.

This distinction between biological activity and clinically meaningful geroprotection should also be considered when interpreting other candidate gerotherapeutics currently undergoing early-phase clinical evaluation.

#### 4.1.3. Statins

Statins are not established geroprotective agents, and their inclusion in this review is based primarily on their extensive human evidence and potential pleiotropic effects on biological processes implicated in aging rather than on demonstrated effects on lifespan or healthspan. In addition to lowering low-density lipoprotein cholesterol and reducing cardiovascular risk, statins may influence inflammation, oxidative stress, endothelial dysfunction, and cellular senescence, providing a plausible mechanistic rationale for potential effects beyond lipid lowering [71,72]. Their particularly extensive clinical evidence also makes statins relevant to the translational framework of this review: they illustrate how an intervention with well-established effects on major age-related disease outcomes may nevertheless lack direct evidence that it modifies the underlying biology of aging. Thus, statins are considered here as a repurposing candidate with potential geroprotective properties, rather than as a clinically validated geroprotector.

Statins, HMG-CoA reductase inhibitors, widely used in the treatment of hypercholesterolemia and cardiovascular prevention, in addition to lipid-lowering effects also exhibit pleiotropic effects—anti-inflammatory and endothelial-protective [71,72]. A multicenter study in an animal model demonstrated that long-term administration of simvastatin to healthy individuals of both sexes was not associated with a significant extension of median overall survival, in contrast to rapamycin administration [16].

Meta-analyses suggest that statins may exert neuroprotective effects and are associated with a lower risk of dementia and reduced all-cause mortality in individuals with elevated cardiovascular risk. However, these findings originate primarily from observational studies and remain susceptible to residual confounding [73].

Statin treatment was significantly associated with a reduced risk of all-cause mortality (RR, 0.92 [95% CI, 0.87–0.98]); stroke (RR, 0.78 [95% CI, 0.68–0.90]); heart attack (RR, 0.67 [95% CI, 0.60–0.75]); and composite cardiovascular events (RR, 0.72 [95% CI, 0.64–0.81]) [70].

Additionally, rosuvastatin demonstrated the most pronounced protective effect against overall dementia among statins (HR 0.72; 95% CI: 0.60–0.88) [74]. At the same time, in some individuals, adverse effects of statins (myopathies, reduction in coenzyme Q10 levels) may paradoxically worsen muscle and mitochondrial function as well as weaken training adaptation [75].

There is also no scientific evidence that statins extend lifespan in healthy individuals with a normal lipid profile. Thus, current evidence supports statins primarily as disease-preventive agents rather than direct geroprotective interventions.

The selection of interventions in this review is not intended to provide an exhaustive catalogue of all compounds proposed in the aging literature, but rather to illustrate pharmacological strategies representing different aging-related pathways, levels of preclinical and clinical evidence, and stages of translational development.

#### 4.1.4. GLP-1R Agonists and SGLT2 Inhibitors

Antidiabetic drugs with documented cardiovascular and nephroprotective benefits—SGLT2 inhibitors and GLP-1 receptor agonists—are also being considered in the context of geroprotection. SGLT2 inhibitors lower blood glucose levels by increasing its excretion in urine, which improves metabolic flexibility.

Moreover, studies conducted in animal models confirmed the senomorphic activity of SGLT2 inhibitors leading to a statistically significant reduction in SASP expression, inhibitory effect on inflammaging and NF-kB/inflammasomes activation, reduced production of free radicals, autophagy regulation, nutrient-sensing pathways, and gut microbiome modulation [76]. Another mechanism of geroprotective action of SGLT2 inhibitors suggested by researchers is the increase in plasma concentration of AICAR (5-aminoimidazole-4-carboxamide-1-β-d-ribofuranoside)—a metabolite activating AMPK [77].

These mechanisms may contribute to the observed reductions in inflammation and to the cardioprotective, nephroprotective, and potentially geroprotective effects reported for SGLT2 inhibitors [78]. Geroprotective activity was evaluated in studies on mouse models, and it was demonstrated that administration of drugs from this group to healthy animals improved median overall survival by 14% (canagliflozin) and 5.9% (empagliflozin), but only in male individuals [79,80,81].

The researchers concluded that the cause of sex specificity remains unclear, but hypothesized that sexual dimorphism may have been associated with suppression of postprandial glucose peaks or androgen-related sensitivity to SGLT2 inhibitors.

The mechanisms underlying the apparent male-specific lifespan extension observed in experimental studies remain unclear. Several hypotheses have been proposed, including sex-related differences in metabolic regulation and renal physiology; however, these mechanisms have not yet been experimentally confirmed [82].

More recently, henagliflozin became the first SGLT2 inhibitor evaluated in a randomized clinical trial specifically addressing aging-related biomarkers. Compared with placebo, treatment was associated with improvements in leukocyte telomere length, inflammatory markers, and several exploratory biomarkers linked to biological aging, including favorable modulation of the IGF-1/IGFBP-3 axis and metabolomic profiles [83].

GLP-1 receptor agonists reduce body weight, improve glycemic control, and decrease cardiovascular risk in individuals with obesity and metabolic disorders [84,85]. Increasing evidence also suggests that GLP-1 receptor signaling may exert anti-inflammatory and potentially neuroprotective effects independent of weight reduction [86].

Experimental studies have demonstrated that low-dose exenatide improved muscle strength, physical performance, and cognitive function in aged mice, accompanied by favorable transcriptomic and metabolomic changes in multiple tissues [87]. However, these findings remain preclinical and should be interpreted cautiously.

Although GLP-1 receptor agonists represent promising candidates for indirect modulation of aging-related metabolic dysfunction, there is currently no direct clinical evidence demonstrating that these drugs extend lifespan or delay aging in otherwise healthy individuals.

Recent human evidence suggesting epigenetic age deceleration with semaglutide originates from a post hoc exploratory analysis of a 32-week randomized clinical trial conducted in adults with HIV-associated lipohypertrophy. Given the post hoc design, modest sample size, short follow-up, and highly specific study population, these findings should be regarded as hypothesis-generating and require confirmation in dedicated prospective studies before any conclusions regarding geroprotective efficacy in the general population can be drawn [88].

#### 4.1.5. Menopausal Hormone Therapy

Some researchers have proposed that appropriately timed menopausal hormone therapy (MHT) may influence selected biological processes associated with aging [89,90]. The established clinical indication for MHT remains the treatment of menopausal symptoms associated with estrogen deficiency.

Menopause is associated with hormonal changes contributing to increased risk of osteoporosis, sarcopenia, cardiovascular disease, metabolic dysfunction, and cognitive impairment. Persistent vasomotor symptoms and declining estrogen levels have also been associated with measurable impairments in quality of life and certain domains of cognitive and behavioral functioning [91,92]. Moreover, evidence from the Women’s Health Initiative suggests that later age at menopause and a longer reproductive lifespan are associated with increased odds of exceptional longevity in women. Women with a reproductive lifespan exceeding 40 years were more likely to survive to the age of 90 years than those with a shorter reproductive lifespan [93].

Estrogen exerts pleiotropic biological effects through ERα, ERβ, and GPER1 signaling pathways and may modulate several hallmarks of aging, including mitochondrial dysfunction, genomic instability, and chronic inflammation [1,84]. However, the extent to which these mechanisms translate into clinically meaningful geroprotective effects remains incompletely understood.

Liu et al., in a population-based retrospective cohort study involving postmenopausal women (n = 117,763) registered in the British biomedical bank (BIOBank), evaluated biological aging discrepancy: chronological age versus biological age [94]. A baseline survey on MHT use and biological aging biomarkers was conducted from March 2006 to October 2010. The data analysis conducted in December 2023 demonstrated that past use of MHT was associated with a smaller biological aging discrepancy than never use of MHT. The age of MHT initiation was of significant importance. Women who initiated MHT at ≤44 years of age had an “older” biomarker profile, whereas those who started MHT after 45 years of age had a “younger” biomarker profile. The Cox model showed that the use of MHT was associated with an approximately 8–9% lower hazard ratio for all-cause mortality compared with women who had never used MHT. However, these results do not prove causality, as the study was observational and subject to potential residual confounding.

### 4.2. Novel Pharmacological Candidates

#### 4.2.1. Senolytics

Senolytics selectively eliminate senescent cells, thereby reducing SASP-mediated tissue dysfunction and chronic inflammation associated with aging. This therapeutic concept has emerged as one of the most promising approaches in geroscience and has been extensively validated in preclinical models. A strong association was also demonstrated between the burden of certain SASP factors in human tissues and inflammation, frailty/decline in physical performance, and functions of certain organs [43].

Oral administration of a senolytic cocktail composed of dasatinib and quercetin to mice once every 2 weeks reduced the number of naturally occurring senescent cells and led to an extension of median post-treatment survival by 36%. This effect also applied to old mice (equivalent to 75–90 years in humans). Moreover, administration of senolytics also alleviated physical dysfunction associated with aging.

In turn, fisetin administered to old mice extended their survival time by 3 months (~10–13%). Meanwhile, the senolytic navitoclax—a Bcl-2 inhibitor—extended median overall survival of obese animals by ~16.7% [45,95,96,97].

#### 4.2.2. NAD^+^ Precursors and Taurine

NAD^+^ is a key coenzyme in human cells, responsible for redox reactions, ATP production, activation of sirtuins, regulation of autophagy, and serving as a substrate for PARP enzymes (poly-ADP-ribose polymerases), which detect and repair DNA damage. Its level in cells decreases significantly with age, even by 50–80%, therefore various interventions aimed at increasing its level in the body have long been investigated.

Dietary intake of NAD^+^ does not increase NAD^+^ levels in blood or tissues due to degradation of this molecule in the gastrointestinal tract [98,99]. In small clinical trials, substances such as nicotinamide riboside (NR) and nicotinamide mononucleotide (NMN) were shown to increase NAD^+^ levels in humans, improving mitochondrial function and insulin sensitivity [99,100].

Singh et al. demonstrated that higher levels of taurine and hypotaurine in blood correlate with lower BMI and waist-to-hip ratio, lower abdominal obesity, and lower levels of C-reactive protein (CRP) in humans. Higher levels of taurine metabolites were correlated with lower incidence of type 2 diabetes and lower glucose levels, higher hemoglobin levels, higher platelet counts, and higher white blood cell counts.

To test whether taurine deficiency contributes to aging, the authors performed a series of complementary studies in animal models [55,101]. In “middle-aged” mice, orally administered taurine (1000 mg/kg body weight/day) increased median lifespan by ~10–12% and extended the expected lifespan of old mice (28 months) by ~18–25%. In taurine-fed mice, improvement in the function of multiple organs was observed: bones, muscles, pancreas, brain, adipose tissue, immune system, and intestines—suggesting improvement in healthspan. In contrast, in “middle-aged” monkeys (primates) receiving oral taurine supplementation (250 mg/kg body weight/day) for 6 months, the following were observed: reduction in body weight and visceral adipose tissue, improvement in insulin sensitivity, reduction in fasting glucose, decrease in levels of inflammatory biomarkers (CRP, TNF-α, IL-6), lower LDL and triglyceride concentrations, increase in HDL, increased lean body mass and higher grip strength, reduction in the number of senescent cells (CD28-/CD57+ T-cells), as well as increased concentrations of metabolites associated with mitochondrial function and DNA repair. The monkey control group received a standard diet without taurine supplementation.

Abud et al., in a small randomized placebo-controlled trial involving 24 women aged 55–70 years, demonstrated that taurine supplementation at 1500 mg/day for 16 weeks prevented the decline in superoxide dismutase activity and significantly reduced markers of oxidative stress. The authors of the study considered this intervention effective in controlling oxidative stress in the aging process [102].

In contrast, Nie et al., in a systematic review of 34 randomized clinical trials published in 2025, concluded that oral taurine supplementation in adults has the potential to realistically improve a range of cardiometabolic risk markers. The most beneficial effects were observed at doses of 1.5–3 g/day. Despite the heterogeneity of the studies included in the systematic review, the results were directionally consistent [103].

However, there is still a lack of long-term randomized clinical trials demonstrating that taurine supplementation extends lifespan or delays age-related diseases in humans.

Despite encouraging preclinical findings, the translational evidence for taurine remains considerably less mature than that for several other interventions discussed in this review. Although experimental studies have reported effects on multiple aging-related processes and human studies have provided preliminary evidence of biological activity, there is currently insufficient clinical evidence to conclude that taurine delays biological aging, prevents age-related multimorbidity, or extends healthspan in humans. Taurine should therefore be regarded as an emerging geroscience candidate rather than a clinically validated geroprotective intervention.

#### 4.2.3. Epigenetic Modulators

According to the Information Theory of Aging—one of the theories explaining the causes of aging—treats aging as a progressive loss of epigenetic information rather than only the accumulation of genetic mutations. Currently, epigenetic alterations are recognized as one of the “hallmarks of aging” and epigenetic reprogramming represents a promising experimental approach for modifying aspects of cellular aging.

Molecules modulating histone deacetylases (HDAC inhibitors) and DNA methyltransferases are being investigated as potential anti-aging interventions.

In vitro experiments confirmed that chemical-induced partial reprogramming (via small molecule cocktails) significantly improves aging hallmarks in aged human fibroblasts [104].

Jo et al., in a 2025 review of studies, summarized that in vivo partial epigenetic reprogramming is capable of epigenetically rejuvenating cells, but is highly organ-specific. Reversal of epigenetic age without complete loss of cellular identity has emerged as a promising experimental strategy for investigating cellular rejuvenation.

Reprogramming competence largely depends on cellular plasticity, the presence/absence of stem cells, the epigenetic landscape of a given tissue, and local signals [105]. Researchers also point out that in the presence of mutations responsible for accelerated aging, mere “rejuvenation” of cells (e.g., through partial reprogramming) does not solve the problem if the mutation continues to act. In the future, in such situations, combining reprogramming with correction of the cause (LMNA editing, antisense, small molecules) will be crucial [106].

Nevertheless, rigorous clinical studies are required to determine both the efficacy and long-term safety of these approaches, particularly given the narrow boundary between partial cellular rejuvenation and uncontrolled cellular reprogramming.

In January 2026, the U.S. Food and Drug Administration (FDA) cleared the Investigational New Drug (IND) application for ER-100, a therapy based on partial epigenetic reprogramming, enabling initiation of the first-in-human Phase I clinical trial in optic neuropathies [107]. The study will involve patients with optic neuropathies (open-angle glaucoma and non-arteritic anterior ischemic optic neuropathy).

ER-100 therapy utilizes three transcription factors (Yamanaka factors: OCT4, SOX2, KLF4), omitting the c-Myc factor in order to reduce the risk of tumorigenesis and preserve cellular identity [108].

In summary, repurposed drugs such as metformin, rapamycin, SGLT2 inhibitors, GLP-1 receptor agonists, and MHT have generally progressed further in clinical evaluation than most novel geroscience candidates, although none has yet demonstrated broadly applicable clinical geroprotection.

Despite its considerable mechanistic interest, epigenetic reprogramming remains a highly experimental approach to geroprotection. Most available evidence derives from cellular and animal models, in which partial reprogramming has been investigated primarily as a strategy to restore aspects of cellular function and tissue homeostasis. Important translational concerns remain, including the potential for loss of cellular identity, aberrant proliferation, tumorigenesis, and tissue-specific responses. Although early-stage studies have generated substantial interest in the possibility of translating partial reprogramming into regenerative or age-modifying therapies, there is currently no clinical evidence demonstrating that epigenetic reprogramming safely delays biological aging or extends healthspan in humans. Accordingly, this approach should be regarded as a promising but highly speculative future therapeutic strategy rather than an emerging clinically validated geroprotector.

#### 4.2.4. Natural Compounds and Integrative Geroprotection

Recent years have also seen growing interest in complementary and integrative approaches to geroprotection, particularly plant-derived compounds and nutritional interventions targeting multiple hallmarks of aging. Unlike conventional pharmacological agents acting predominantly through a single pathway, many natural compounds exert pleiotropic effects involving AMPK activation, autophagy induction, mitochondrial homeostasis, attenuation of inflammaging, and modulation of oxidative stress. Nevertheless, despite encouraging mechanistic data, their clinical translation remains considerably less robust than preclinical evidence.

Among currently investigated compounds, spermidine and urolithin A possess the strongest translational rationale [109,110]. Spermidine has been associated with enhanced autophagy and modest improvements in cognitive function [111], whereas urolithin A improves mitochondrial function and skeletal muscle performance in older adults [112]. However, neither intervention has yet demonstrated convincing evidence for delaying multimorbidity or extending lifespan in humans [113,114]. Likewise, polyphenols such as epigallocatechin gallate (EGCG), curcumin, quercetin, resveratrol, and fisetin influence multiple longevity-associated pathways, including SIRT1, AMPK, NF-κB, and mitochondrial signaling [115,116,117], but their clinical usefulness remains limited by low bioavailability, heterogeneous study designs, and inconsistent reproducibility across human trials [118,119].

Recent reviews further suggest that selected phytochemicals may exert beneficial effects on immunosenescence, mitochondrial metabolism, and chronic low-grade inflammation, particularly when combined with dietary interventions and healthy lifestyle measures [120,121]. However, these approaches should currently be regarded as supportive rather than established geroprotective therapies, since validated biological-age endpoints and large randomized clinical trials remain scarce [65,122]. Future studies should prioritize standardized biomarkers of biological aging, optimization of formulation and bioavailability, and rigorous clinical validation before natural compounds can be recommended as evidence-based geroprotective interventions.

Despite decades of intensive investigation, most natural polyphenols have not demonstrated reproducible clinical benefits on validated aging outcomes. Limited bioavailability, heterogeneous formulations, and insufficient target engagement remain major obstacles to clinical translation. Consequently, natural compounds should currently be considered complementary rather than evidence-based primary geroprotective interventions.

Overall, current evidence indicates that mechanistic plausibility frequently exceeds clinical validation. While numerous compounds influence conserved pathways implicated in aging, only a limited number have progressed to randomized clinical evaluation, and none has yet demonstrated definitive extension of human lifespan. This discrepancy highlights the importance of distinguishing biological activity from clinically meaningful geroprotective efficacy.

The history of many natural compounds illustrates that repeated promising experimental findings have frequently failed to produce convincing clinical evidence despite decades of investigation.

Future studies should prioritize standardized formulations, pharmacokinetic optimization, target engagement analyses, and reproducible clinical outcomes rather than further expanding mechanistic observations alone.

**Figure 1:**
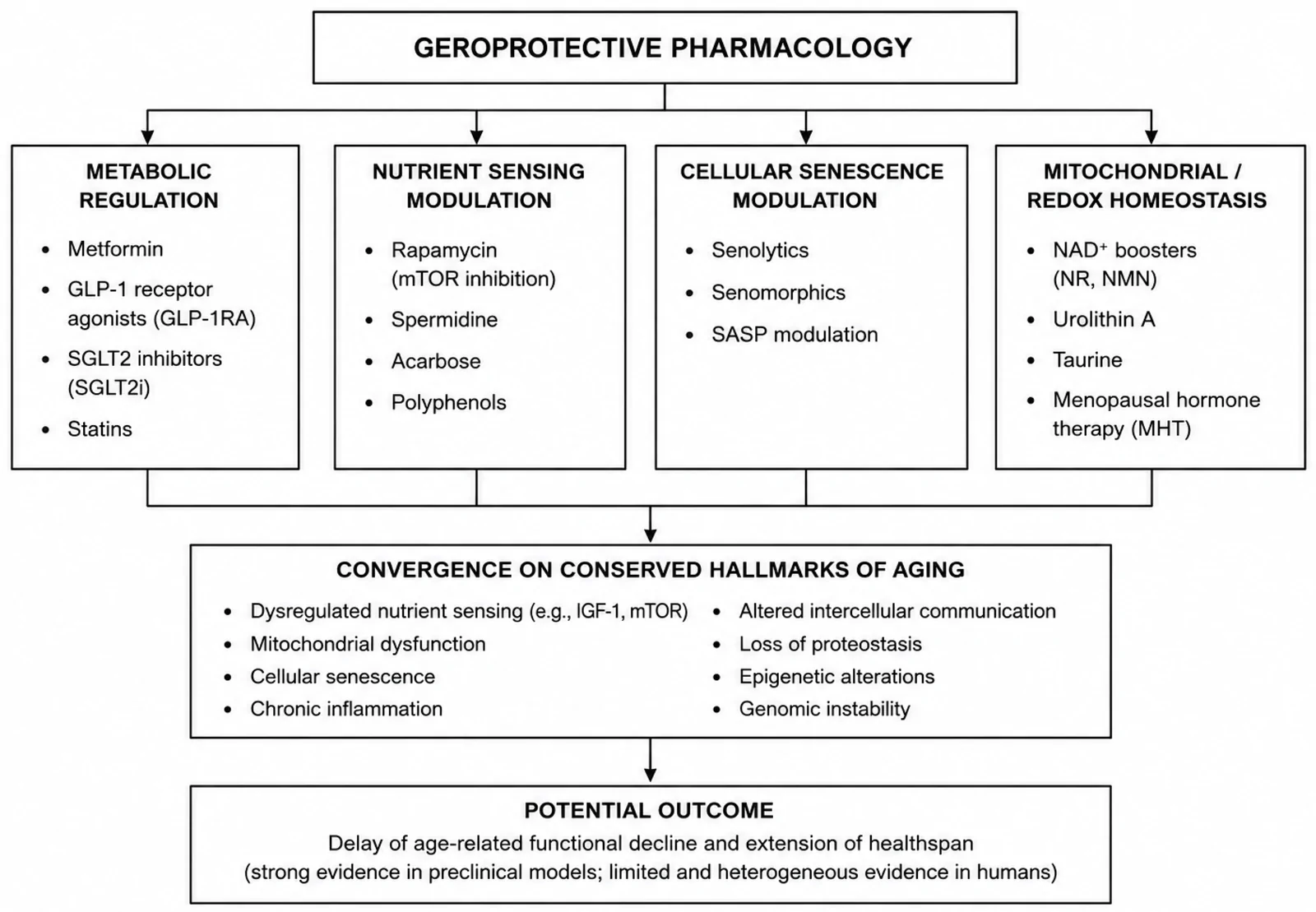
Overview of the principal classes of candidate geroprotective interventions and their major biological targets. Candidate pharmacological and nutritional interventions converge on several conserved biological mechanisms involved in aging, including nutrient sensing, mitochondrial homeostasis, and cellular senescence. Despite extensive preclinical evidence, translation into clinically proven improvement of human healthspan remains limited. Abbreviations: GLP-1RA, glucagon-like peptide-1 receptor agonists; MHT, menopausal hormone therapy; NAD^+^, nicotinamide adenine dinucleotide; SGLT2i, sodium–glucose cotransporter-2 inhibitors.

To facilitate interpretation of the complex mechanisms discussed throughout this review, Figure 1 summarizes the principal classes of candidate geroprotective interventions together with their major biological targets and their relationship to conserved hallmarks of aging.

## 5. Clinical Evidence from Human Studies

Although most evidence for the geroprotective effects of drugs comes from animal studies, the number of clinical and observational studies testing potential longevity therapies in humans is increasing. This area remains limited, but several interventions deserve particular attention.

### 5.1. Metformin: TAME and Further Directions

The most ambitious clinical initiative evaluating geroprotection is the planned TAME (Targeting Aging with Metformin) trial involving more than 3000 individuals aged 65–80 years without diabetes, which is planned to last 6 years [59,123]. The trial is designed to determine whether metformin can delay the development of multiple age-related diseases (cardiovascular events, cancer, cognitive decline), rather than evaluating only a single endpoint, which constitutes an innovative approach in the design of clinical studies on aging.

Observational studies provide support: patients initiating metformin therapy demonstrated lower all-cause mortality and lower risk of diseases of older age than individuals treated with sulfonylureas or healthy individuals [19,21,60,124,125]. Importantly, observational associations should not be interpreted as proof of geroprotective efficacy, since they remain susceptible to residual confounding, healthy-user bias, and indication bias. Meta-analyses confirm protective associations of metformin with reduction in the risk of cancer and cardiovascular diseases, although causality remains uncertain [126].

However, despite several years of announcements, the TAME study has still not been initiated according to the original plan due to problems with its funding (the American Federation for Aging Research continues fundraising to support and launch the TAME study) [106,127].

On the other hand, the recent double-blind, randomized, placebo-controlled MET-PREVENT study, which aimed to assess the effect of metformin on physical performance in older individuals diagnosed with probable sarcopenia or frailty syndrome, did not meet its endpoint [128]. The primary endpoint of the study was the change in 4-m walk speed from baseline, adjusted for initial values. The study population was small (n = 36) and consisted of very old adults (mean age 80.4 years) with established frailty or probable sarcopenia, in whom advanced biological aging may have limited the effectiveness of metabolic interventions. The average age of participants was as high as 80.4, even though the study lasted only 4 months. In the study group, mitochondrial dysfunction, high burden of senescent cells, and chronic systemic inflammation may already have been too advanced to be reversed by metabolic modulation alone.

These findings also support a more cautious interpretation of metformin as a geroprotective intervention. Although observational studies and experimental models suggest beneficial effects on multiple aging-related pathways, recent randomized clinical evidence indicates that these benefits may not be generalizable across all older populations. Rather than representing a universal geroprotector, metformin may ultimately prove to be most effective in selected subgroups or when initiated earlier in the aging trajectory, before advanced frailty and irreversible tissue dysfunction become established. Future biomarker-guided trials will be essential to identify individuals most likely to benefit from metformin-based geroprotection.

The discrepancy between observational studies and randomized clinical trials further emphasizes that associations with reduced mortality should not be interpreted as proof of causal geroprotective activity.

Concluding, although metformin remains the most extensively investigated geroprotective candidate, recent randomized clinical studies have produced mixed results, indicating that its effects may depend on patient selection, age, metabolic status, and treatment duration. These findings highlight the need for larger, well-designed clinical trials using clinically meaningful aging-related endpoints before routine geroprotective use can be recommended.

### 5.2. Rapamycin and Rapalogs

Small clinical studies suggest that rapamycin and its analogs may modulate aging processes. A randomized study of low-dose everolimus in older individuals improved the response to influenza vaccination, suggesting “rejuvenation” of the immune system [13]. Short-term use of rapamycin reduced the expression of aging markers in the skin, such as p16INK4a [129].

In 2023, Kaeberlein et al. described the effects of rapamycin use in a community of 333 healthy older individuals (mean age approximately 61 years) for “healthy longevity/anti-aging.” The most commonly used dose was 3–6 mg once weekly.

The authors reported that participants receiving rapamycin exhibited a significantly lower probability of COVID infection and development of long COVID. These individuals also reported subjective improvement in quality of life, including various indicators of well-being and physical endurance.

Apart from mild oral ulcers (mouth sore), rapamycin users did not report any safety signals. A limitation of this study is the lack of blinding; therefore, the placebo effect may have played a certain role [130].

Kraig et al. evaluated in a small clinical study the effect of rapamycin use in healthy older adults (aged 70–95 years), at a dose of 1 mg/day for 8 weeks. The study did not demonstrate significant improvement in various metabolic parameters, apart from reduction in body weight [131]. However, a later analysis of the data using the biomarker-based aging clock “PhenoAge” demonstrated that individuals receiving rapamycin reduced their phenotypic age by an average of approximately 4 years compared with +0.15 years in the placebo group [132].

In 2025, the results of the PEARL trial were published, the largest and longest randomized clinical trial of intermittent low-dose rapamycin in generally healthy middle-aged and older adults evaluating its safety and potential effects on healthspan-related outcome. This study demonstrated that administration of compounded rapamycin at doses of 5 mg/week or 10 mg/week (equivalent to approximately 1.4 and 2.9 mg of standard sirolimus), intermittently for 48 weeks, is relatively safe in generally healthy middle-aged and older individuals. The higher-dose regimen was associated with modest improvements in muscle mass and self-reported pain among women. Women using 5 mg/week reported improvement in subjective assessment of health status (General Health) and improvement in emotional well-being [133].

Although the PEARL trial demonstrated an acceptable short-term safety profile and modest improvements in selected secondary outcomes, including muscle mass and self-reported pain in women, it did not demonstrate significant improvements in several predefined aging-related clinical endpoints, including visceral adiposity. Therefore, the observed benefits should be interpreted as exploratory rather than definitive evidence of geroprotection.

Additional clinical trials evaluating rapamycin in the context of cardiovascular health, immunosenescence, and delay of menopause are ongoing or were recently completed, but full data are not yet available [134,135,136,137].

Despite numerous preclinical and preliminary clinical data confirming the usefulness of rapamycin and other mTOR inhibitors as potential gerotherapeutics, at the time of preparation of this paper, human studies had still not demonstrated that rapamycin can extend average or maximal lifespan or delay the onset of age-related diseases [95].

A recent systematic review emphasized that there is currently no clear clinical evidence that rapamycin or its derivatives safely slow biological aging or extend lifespan in humans. While available studies suggest biological activity and acceptable tolerability at low intermittent doses, the overall certainty of evidence remains limited because of small sample sizes, heterogeneous endpoints, and relatively short follow-up periods [138].

Accordingly, rapamycin should currently be regarded as an experimental gerotherapeutic with strong mechanistic rationale but limited clinical confirmation of efficacy in healthy humans.

Collectively, available randomized studies indicate acceptable short-term safety but insufficient evidence for routine preventive use. Current clinical data support continued investigation rather than implementation in healthy populations.

### 5.3. Senolytics

Senolytic therapies have now entered early-phase clinical evaluation in humans. To date, fewer than ten clinical studies evaluating senolytics have been published, and only a minority have included randomized controlled designs. Consequently, current human evidence remains insufficient to determine whether senolytic therapy can modify biological aging beyond disease-specific effects.

In a pilot open-label study, dasatinib with quercetin, administered to patients with idiopathic pulmonary fibrosis, reduced biomarkers of senescence and improved physical performance [30,51,139]. In another study involving patients with chronic kidney disease, reduction in senescence-associated transcripts and SASP markers in blood was observed [140].

In an open-label, randomized phase II study involving 60 healthy postmenopausal women (aged 62–88 years), administration of dasatinib (100 mg/day for 2 consecutive days every 28 days) with quercetin (1000 mg/day for 3 consecutive days every 28 days) for 20 weeks led to improvement in bone BMD, but exclusively in the group with the highest senescent cells burden, as assessed by the T cell p16 assay (exploratory analyses). The study confirmed the hypothesis that the skeletal response to senolytics depends on the existing burden of senescent cells [141].

This suggests an important role of personalization of senolytic treatment based on aging biomarkers. Currently, several clinical studies are being conducted to evaluate the effectiveness of senolytics in the treatment of disease states or aging-related biomarkers such as frailty, inflammatory markers, insulin sensitivity, senescence markers, and knee osteoarthritis [142,143,144].

Recent phase II studies in Alzheimer’s disease (including the SToMP-AD and STAMINA programs) further illustrate both the promise and the current limitations of senolytic therapy. Although these trials provide valuable safety and feasibility data, they have not yet demonstrated definitive disease-modifying or geroprotective effects, underscoring the need for larger randomized studies with clinically meaningful aging endpoints [145,146,147].

These findings also emphasize the biological heterogeneity of cellular senescence. Senescent cell burden differs substantially between individuals, tissues, and disease states, suggesting that senolytic therapies are unlikely to provide uniform clinical benefit. Rather than treating chronological aging as a homogeneous condition, future clinical trials will likely need to stratify participants according to validated biomarkers of cellular senescence.

The exploratory analysis reported by Farr et al. further suggests that baseline senescent cell burden may determine therapeutic responsiveness, supporting this precision-medicine approach in future senolytic trials [141,148,149].

Collectively, these findings indicate that senolytic therapies are unlikely to provide universal clinical benefit, and their success will probably depend on identifying the patients most likely to respond. Despite encouraging biological signals, the available clinical evidence remains limited. To date, only a small number of interventional clinical studies have been completed, and only a minority included randomized controlled designs. Most trials evaluated biomarker changes or disease-specific outcomes rather than validated measures of biological aging, limiting conclusions regarding true geroprotective efficacy.

The use of senolytics is also being investigated in the context of accelerated aging in specific populations. An example of such a study is the SEN-SURVIVORS Trial—the first-ever open-label intervention trial evaluating the effectiveness of senolytics in reducing senescence and improving frailty in adult survivors of childhood cancer [150].

Overall, senolytics represent one of the most biologically compelling approaches in geroscience. Nevertheless, current human evidence remains preliminary and should be interpreted cautiously until larger randomized trials establish reproducible clinical benefits beyond biomarker modulation.

### 5.4. NAD^+^ Precursors

Supplementation with nicotinamide riboside (NR) and nicotinamide mononucleotide (NMN) has been evaluated in small clinical trials. Chronic administration of NR increased NAD^+^ levels in healthy older individuals and was well tolerated [73]. Administration of NMN improved insulin sensitivity and muscle metabolism in overweight individuals or those with prediabetes [100].

Kuerec et al. demonstrated that after 60 days of NMN supplementation in a group of healthy middle-aged individuals, the increase in blood NAD^+^ levels was associated with improvement in red blood cell parameters, potentially indicating enhanced oxygen transport capacity [151].

However, the studies were short-term and did not evaluate “hard” aging-related endpoints. Two recently published systematic reviews suggest lack of effect of NMN supplementation on fasting glycemia, triglycerides, TC, LDL-C, HDL-C, and lack of significant improvement in skeletal muscle index, handgrip strength, gait speed, 5-time chair stand test, and 6-min walking distance test [152,153].

In turn, a meta-analysis of 9 studies published in 2025 demonstrated that NMN has positive efficacy in enhancing muscle function, reducing insulin resistance, and lowering hepatic aminotransferase levels in middle-aged and elderly individuals. The researchers also noticed that the majority of studies assessed lower doses of NMN and larger doses may lead to more significant improvements in gait speed in middle-aged and elderly people. At the same time, the authors of the meta-analysis emphasized that limitations related to the quality, heterogeneity, and small sample size of the included studies should be taken into account, and further long-range clinical trials are required to evaluate the influence of NMN on muscle and liver functions in middle-aged and elderly populations [154].

Due to the favorable safety profile, an increasing number of countries permit the sale of NR and NMN as dietary supplements or complementary medicines [155,156,157,158].

### 5.5. GLP-1R Agonists and SGLT2 Inhibitors

GLP-1 receptor agonists (GLP-1RA) and SGLT2 inhibitors, although not directly investigated as anti-aging therapies, demonstrated reduction in cardiovascular mortality in non-diabetic individuals and reduction in kidney disease progression in large clinical studies [84,159,160]. Their pleiotropic effects—on body weight, inflammation, and vascular function—make them potential indirect geroprotective factors. It is worth emphasizing that GLP-1 agonists exert clinical benefits even in individuals who do not experience significant weight loss [161]. Thus, GLP-1RA represent promising candidates for indirect modulation of aging-related metabolic dysfunction, although definitive geroprotective effects in humans remain unproven.

Corley et al. published in 2025, for the first time, data from a randomized clinical trial suggesting that semaglutide modulates validated epigenetic biomarkers of aging in adult individuals with HIV-associated lipohypertrophy. These results signal a possible geroprotective effect, but the study is burdened by significant limitations: the conclusions were based on post hoc biomarker analysis, the study was conducted in a specific patient population, and over a short time horizon of 32 weeks [162].

Accordingly, these findings should be regarded as hypothesis-generating rather than confirmatory. The observed reduction in epigenetic age was derived from a post hoc analysis performed in a highly selected population with HIV-associated lipohypertrophy and cannot currently be extrapolated to healthy individuals or interpreted as evidence of primary geroprotection.

Henagliflozin, a novel SGLT2 inhibitor, has recently become the first agent in this class to demonstrate favorable effects on validated biomarkers associated with biological aging in a randomized clinical trial, including increased leukocyte telomere length and beneficial changes in IGFBP-3, β-hydroxybutyrate, and selected immune biomarkers. Although these findings are encouraging, they should be interpreted cautiously until replicated in larger studies with clinical aging endpoints [83,114,122,163].

At the time of completion of this paper, no studies conducted in healthy participants with primary geroprotective endpoints (e.g., time to disability, frailty progression, multimorbidity, and mortality independent of baseline diseases) had been published. There is a lack of hard scientific evidence for extension of healthspan/lifespan in humans through preventive use of GLP-1RA. Potential use of SGLT2 inhibitors for prevention of age-related diseases in healthy individuals requires further studies.

Therefore, current evidence supports the use of GLP-1RA primarily for their established cardiometabolic benefits. Any direct anti-aging effect in humans remains speculative and requires confirmation in randomized trials specifically designed to evaluate biological aging outcomes. It also remains uncertain to what extent the observed biological benefits result from direct receptor-mediated mechanisms rather than secondary effects of weight reduction and improved metabolic control.

In summary, current clinical evidence supports substantial cardiometabolic benefits of GLP-1RA and SGLT2 inhibitors in appropriately selected patients. However, direct evidence that these agents slow biological aging or extend healthspan in otherwise healthy individuals remains insufficient. Their potential role as geroprotective therapies should therefore be considered promising but still unproven.

### 5.6. Emerging Geroprotective Compounds

Several additional pharmacological and nutraceutical interventions have recently emerged as potential geroprotective candidates. Among these, spermidine, urolithin A, and acarbose have generated increasing interest because they target complementary mechanisms involved in aging biology, including autophagy, mitochondrial quality control, and glucose metabolism.

Among dietary geroprotective candidates, spermidine currently possesses one of the strongest mechanistic rationales owing to its ability to induce autophagy. Nevertheless, available human studies remain relatively small and focus mainly on cognitive or metabolic outcomes rather than validated aging endpoints [155,164].

Spermidine stimulates autophagy through inhibition of EP300 acetyltransferase activity [165]. It has demonstrated favorable effects on cognitive performance and cardiovascular risk markers in early clinical studies [166]. However, current human evidence remains insufficient to conclude that spermidine slows biological aging or extends healthspan [155,167].

Urolithin A enhances mitophagy and mitochondrial function and has shown improvements in muscle endurance and mitochondrial biomarkers in randomized clinical trials involving older adults [112,168,168]. Nevertheless, available studies remain relatively small and have not yet demonstrated reductions in age-related morbidity or mortality.

Acarbose, an α-glucosidase inhibitor, has consistently extended lifespan in genetically heterogeneous mice, particularly in males [169], and is considered one of the most reproducible pharmacological interventions identified by the National Institute on Aging Interventions Testing Program [22,170,171]. However, clinical evidence supporting geroprotective effects in humans remains lacking.

### 5.7. Menopausal Hormone Therapy

Geroprotection based on MHT still raises many controversies. Both animal studies and randomized controlled clinical trials clearly confirm that MHT is significantly more effective in maintaining vascular health (healthy endothelium) than in treating developed vascular disease manifested by atherosclerotic lesions [90,172]. These data reinforce the importance of the timing of MHT initiation.

A comparison of younger women (aged 50–59 years) and older women (aged 70–79 years) in the pooled Women’s Health Initiative (WHI) Estrogen-Plus-Progestin and Estrogen-Alone Trials showed that the ratio of the nominal HRs for all-cause mortality was 0.61 (95% CI, 0.43–0.87) during the intervention phase, and 0.87 (95% CI, 0.76–1.00) during the 18-year cumulative follow-up. There was no significant heterogeneity between the trials [173].

A meta-analysis of 19 randomized controlled trials involving a total of 40,410 postmenopausal women using MHT (mostly orally) did not demonstrate a significant increase in all-cause mortality or cardiovascular mortality in women using MHT [174].

The subgroup analysis based on the timing of MHT initiation demonstrated that women who initiated MHT within 10 years after menopause had lower mortality (RR, 0.70 [95% CI, 0.52–0.95]) and fewer cardiac events (RR, 0.52 [95% CI, 0.29–0.96]). In contrast, women who initiated MHT more than 10 years after menopause onset demonstrated increased risk of stroke. At the same time, in both groups, regardless of the timing of MHT initiation, the researchers observed an increase in thromboembolic events.

The interpretation of the WHI findings has been extensively debated, particularly regarding participant age, timing of therapy initiation, and hormone formulation. Contemporary evidence suggests that initiation of MHT before the age of 60 years or within 10 years after menopause onset may have a more favorable benefit-risk profile in appropriately selected women [90,173,174,175,176,177]. Nevertheless, major scientific societies currently do not recommend MHT solely for the prevention of age-related diseases in asymptomatic women [178,179,180,181].

Table 1, Table 2 and Table 3 systematize the presented knowledge and provide tabular summaries and conceptual schemes that are useful for visualization of pharmacological targets, clinical evidence, and translational challenges in geroprotective medicine.

Taken together, these studies illustrate the heterogeneous translational maturity of geroscience interventions. Clinical investigation has progressed from mechanistic and biomarker-oriented studies toward randomized trials assessing functional and disease-related outcomes, but no intervention has yet established broadly applicable pharmacological geroprotection in humans. Importantly, biological activity or improvement in an individual aging-related endpoint should not be equated with delayed multimorbidity or extended healthspan. The current clinical landscape therefore highlights the persistent gap between modulation of aging-associated pathways and demonstration of clinically meaningful geroprotection.

Importantly, interventions with relatively strong clinical evidence for disease-specific benefits, such as SGLT2 inhibitors, GLP-1 receptor agonists, and statins, should not be interpreted as clinically validated geroprotectors, because their randomized trials have generally targeted specific diseases or risk factors rather than validated measures of biological aging, healthspan, or delayed multimorbidity.

An important lesson emerging from contemporary geroscience is that favorable modulation of molecular pathways does not necessarily translate into clinically meaningful slowing of aging. Several interventions that consistently improved lifespan or healthspan in experimental models have subsequently demonstrated neutral or inconclusive findings in randomized human studies. This translational gap highlights the complexity of human aging and reinforces the need for validated biological aging biomarkers, appropriate patient selection, and clinically relevant endpoints [63,64,65,182].

**Figure 2:**
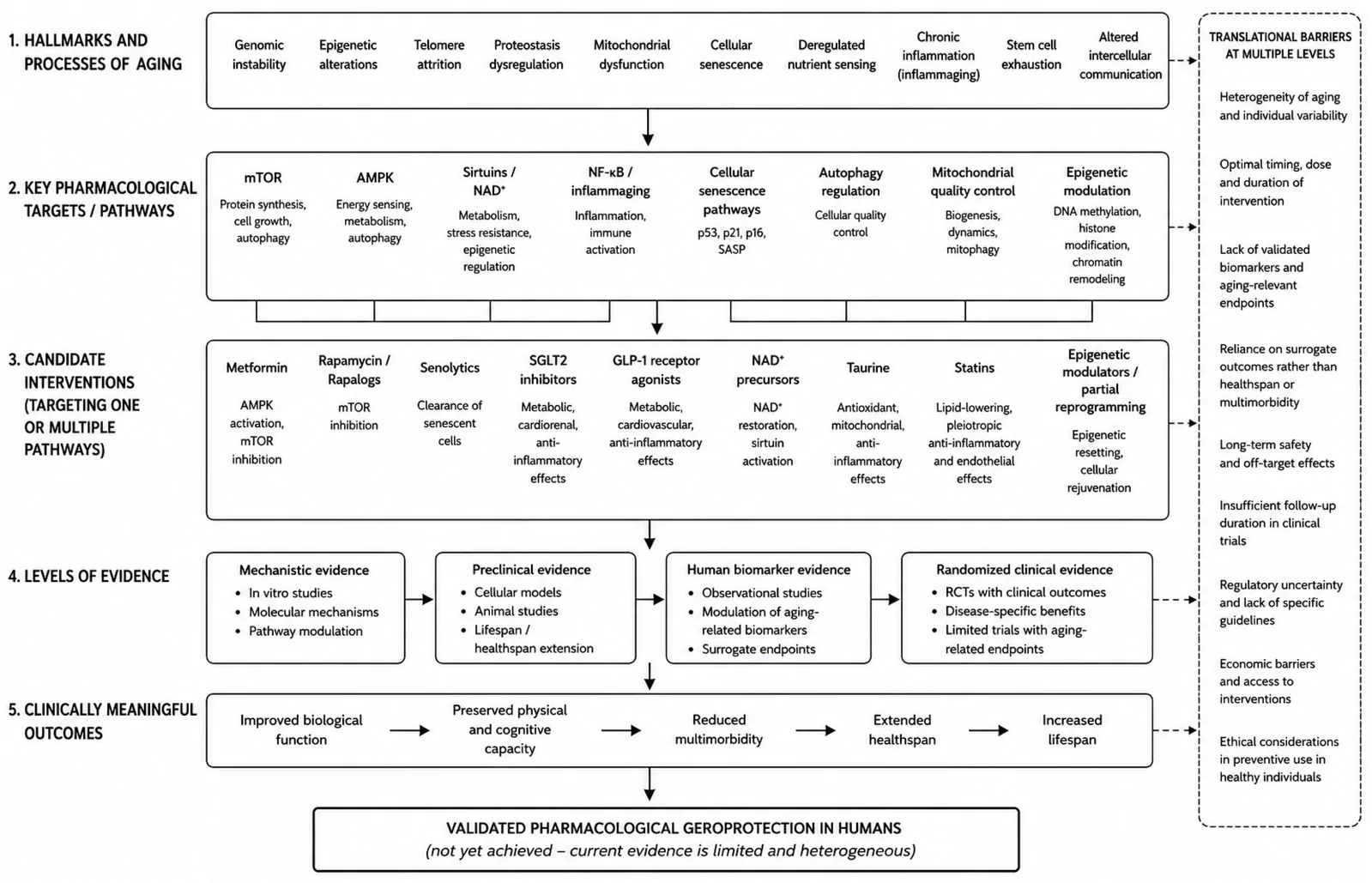
From aging hallmarks to clinical geroprotection: a translational framework.

Taken together, the evidence reviewed above illustrates a substantial gap between mechanistic plausibility and clinically validated geroprotection. Candidate interventions can be positioned along a translational continuum that extends from modulation of aging-related pathways and supportive preclinical findings to human biomarker studies and randomized clinical trials assessing clinically meaningful outcomes. However, progression through these stages is not linear, and strong evidence at one level does not necessarily predict successful translation to the next. Biological heterogeneity, limitations of aging biomarkers and surrogate endpoints, uncertainty regarding treatment timing and duration, long-term safety considerations, and regulatory and economic barriers may all contribute to attrition during clinical development. This translational framework is summarized in Figure 2 and highlights why promising geroscience targets may remain clinically unvalidated despite substantial mechanistic and preclinical support.

Figure 2 illustrates the progression from biological hallmarks and aging-related pathways through candidate pharmacological interventions and increasing levels of evidence toward clinically meaningful outcomes. Translational progression is constrained by biological heterogeneity, limitations of aging biomarkers and surrogate endpoints, intervention timing and duration, long-term safety, insufficient clinical follow-up, regulatory and economic barriers, and ethical considerations. RCTs, randomized controlled trials; SASP, senescence-associated secretory phenotype.

## 6. Translational Barriers to Clinically Validated Geroprotection

Despite remarkable progress in understanding the molecular biology of aging, successful translation into effective human geroprotective therapies has remained limited. Several factors contribute to this discrepancy, including biological differences between short-lived experimental organisms and humans, the multifactorial nature of aging, heterogeneity of study populations, differences in treatment timing, insufficient treatment duration, and the absence of validated clinical aging endpoints. Consequently, interventions demonstrating robust mechanistic activity should not automatically be considered clinically effective geroprotectors [63,64,65,182].

Although numerous interventions demonstrate favorable biological effects in experimental models, successful translation into clinical geroprotection remains limited by the complex interaction of biological, methodological, regulatory, and ethical barriers. The central translational challenge is therefore not simply to identify compounds capable of modifying individual aging-associated pathways, but to demonstrate that such biological effects produce durable and clinically meaningful improvements in human healthspan.

### 6.1. Biological and Population-Level Barriers

A large proportion of the evidence supporting candidate geroprotective interventions originates from preclinical studies conducted in short-lived animal models maintained under highly controlled laboratory conditions. Translation of these findings into humans is challenging because of substantial interspecies differences in metabolism, lifespan regulation, immune function, microbiome composition, environmental exposures, and the organization of age-related pathology. Aging in humans is also a highly heterogeneous and multifactorial process, making it unlikely that modulation of a single molecular pathway will produce uniform effects across populations.

Population heterogeneity further complicates translation. Individuals enrolled in clinical studies may differ substantially with respect to biological age, metabolic phenotype, genetic background, comorbidity burden, lifestyle, and baseline risk of age-related disease. These factors may influence both treatment response and the clinical relevance of a given geroprotective mechanism. Consequently, future therapeutic strategies may require greater stratification according to biological age, metabolic phenotype, burden of cellular senescence, genetic background, and disease-specific risk profiles. Biomarker-guided precision geroprotection may therefore become an important component of successful clinical translation.

Differences in treatment timing represent another potential barrier. An intervention that is effective when initiated early in the aging process may have substantially different effects when administered after extensive accumulation of cellular damage or established multimorbidity. Similarly, treatment durations used in clinical studies may be insufficient to detect changes in long-term aging-related outcomes. These considerations emphasize the importance of appropriately selected populations, treatment windows, and sufficiently long follow-up periods.

### 6.2. Biomarkers and Clinical Endpoints

Another major obstacle is the absence of universally accepted biomarkers capable of accurately measuring biological aging and therapeutic response. In contrast to classical clinical studies based on disease-specific endpoints, trials of geroprotective drugs require reliable and validated measures capable of capturing changes in the biology of aging. Current approaches, including epigenetic clocks, circulating SASP components, inflammatory markers, proteomic signatures, and functional assessments, remain incompletely validated for regulatory decision-making and routine clinical practice [183,184].

Reliable biomarkers of biological aging are nevertheless essential for evaluating geroprotective interventions, and substantial progress has been made in recent years toward their development and standardization [183]. Without adequate validation and standardization, however, it remains difficult to determine whether a biomarker reflects a clinically meaningful change in aging biology, to compare interventions across studies, or to use biomarker modulation as evidence of therapeutic efficacy.

The absence of standardized clinical endpoints creates a related problem. Most existing trials report disease-specific outcomes, isolated biomarkers, or relatively short-term functional measures rather than validated measures of biological age, resilience, frailty, multimorbidity, disability-free survival, or mortality. Consequently, a change in an aging-associated biomarker should not be interpreted as equivalent to demonstrated improvement in human healthspan. The development and validation of clinically meaningful geroscience endpoints therefore remain central priorities for translational research.

### 6.3. Study Design, Reproducibility, and Evidence Quality

Most currently available clinical trials evaluating geroprotective interventions are characterized by relatively small sample sizes, short duration, and reliance on surrogate biomarkers rather than clinically meaningful endpoints. Such designs may be appropriate for establishing biological activity or preliminary safety, but they are generally insufficient to demonstrate durable effects on healthspan or age-related morbidity.

Human evidence is also frequently derived from observational studies, which are susceptible to residual confounding, healthy-user bias, and indication bias. Individuals receiving medications such as metformin, statins, GLP-1 receptor agonists, or menopausal hormone therapy may differ substantially from untreated populations with respect to socioeconomic status, healthcare access, lifestyle, and baseline health status. Accordingly, observational associations should be interpreted primarily as hypothesis-generating rather than as evidence of causal geroprotection.

Reproducibility represents an additional challenge. Several interventions that initially generated considerable enthusiasm have subsequently produced inconsistent or difficult-to-reproduce findings across independent laboratories and clinical settings. This underscores the importance of rigorous experimental design, independent validation, transparent reporting, and adequately powered randomized clinical trials. Publication bias and commercial interests may further contribute to overrepresentation of positive findings, particularly in rapidly developing fields such as NAD^+^ precursors, senolytics, and epigenetic reprogramming. Positive preclinical studies are more likely to be published than neutral or negative findings, potentially leading to overestimation of geroprotective efficacy during early stages of drug development.

Overall, these limitations indicate that mechanistic plausibility and favorable preclinical findings should be regarded as necessary but insufficient steps in the development of geroprotective therapies. Most proposed interventions should currently be regarded as investigational until their efficacy and long-term safety are confirmed in adequately powered randomized clinical trials employing validated biomarkers and clinically meaningful aging-related endpoints.

### 6.4. Long-Term Safety and Tolerability

Most candidate geroprotective drugs were originally developed for the treatment of specific diseases rather than for long-term administration to otherwise healthy individuals [27,28]. This distinction is particularly important because geroprotective interventions may ultimately be administered for prolonged periods to individuals who are not currently suffering from the disease for which a drug was originally developed.

Metformin is generally well tolerated, but may cause mild gastrointestinal adverse effects and, rarely, lactic acidosis in patients with renal insufficiency [31]. Senolytics such as dasatinib or navitoclax may induce hematological toxicity and off-target effects, which complicates their safe use [48]. In contrast, plant-derived senolytics, NAD^+^ precursors, and taurine are characterized by a favorable safety profile, although their suitability for long-term geroprotective use requires continued clinical evaluation. The development of safe long-term dosing regimens therefore remains one of the major translational challenges.

Long-term safety is particularly important when interventions are considered for prevention rather than treatment of established disease. Even modest adverse effects may become clinically relevant when exposure extends over many years. Accordingly, future geroscience trials should incorporate systematic assessment of adverse events, treatment discontinuation, organ-specific toxicity, drug interactions, and long-term tolerability.

### 6.5. Regulatory, Economic, and Ethical Barriers

The clinical development of geroprotective therapies is further complicated by regulatory and economic considerations. Aging itself is not currently recognized as a disease by most regulatory agencies, which substantially complicates the clinical development and approval of geroprotective therapies. However, the introduction of the MG2A code into the ICD-11 classification represents a significant step forward in the area of designing and evaluating clinical studies. Conducting clinical studies aimed at evaluating delay of multimorbidity requires long follow-up, large cohorts, and significant budgets [185,186].

For naturally derived substances or synthetic substances not qualifying for patent protection, such studies may be difficult to justify economically. Large studies financed by governmental organizations also encounter substantial funding constraints [59,187]. Regulatory frameworks must therefore continue to evolve so that clinical trials aimed at evaluating therapies with geroprotective activity become more economically feasible for industry while maintaining rigorous standards of evidence [188,189].

The development of pharmacological geroprotective therapies also raises ethical, social, and economic questions [190]. One concern is the potential medicalization of natural aging, which could shift societal attention from lifestyle and environmental determinants of healthy aging toward pharmacological interventions [3]. Questions regarding the optimal timing of intervention and equitable access across aging populations will also require consideration.

The longevity field has additionally faced concerns regarding exaggerated scientific claims, premature commercialization, and selective interpretation of preliminary findings. Many compounds are widely promoted despite limited human evidence, creating a substantial gap between biological plausibility and demonstrated clinical efficacy. Independent replication, transparent reporting, and prioritization of high-quality randomized clinical trials are therefore essential before widespread implementation of geroprotective interventions.

These considerations emphasize the importance of distinguishing clearly between mechanistic hypotheses, biomarker modulation, and clinically validated geroprotective effects. Successful implementation of geroprotective therapies will require not only scientific progress, but also appropriate regulatory frameworks, equitable access, rigorous clinical validation, and responsible communication of scientific evidence. Avoiding unrealistic expectations generated by premature commercialization of interventions supported only by preliminary mechanistic or preclinical evidence is equally important.

### 6.6. Strategies to Facilitate Clinical Translation

The translational gap may be reduced by adopting clinical strategies that connect mechanistic hypotheses more directly with meaningful human outcomes. One approach involves evaluating candidate geroprotective mechanisms in age-related diseases with well-defined clinical endpoints. Idiopathic pulmonary fibrosis, a disease strongly driven by fundamental mechanisms of biological aging, provides an attractive clinical setting for evaluating geroscience-based interventions, and the development of the TNIK inhibitor rentosertib illustrates how disease-oriented clinical trials may accelerate translation into clinical practice [1,186,191,192].

Future clinical studies should increasingly integrate validated biomarkers with functional and clinical outcomes rather than relying on biomarker modulation alone. Trials should ideally incorporate measures of multimorbidity, physical and cognitive function, frailty, disability-free survival, or other clinically meaningful outcomes alongside molecular measures of aging. This approach may provide a more robust framework for determining whether modification of an aging-associated pathway translates into meaningful improvements in human health.

The translational pathway should therefore be viewed as a progression from mechanistic plausibility through reproducible preclinical efficacy, human biological activity, and biomarker validation to clinically meaningful outcomes. Failure to demonstrate benefit at any of these stages should prompt reassessment of the underlying target, intervention, population, or endpoint rather than being interpreted solely as a failure of clinical implementation.

## 7. Future Research Directions

Future geroprotection research should increasingly focus on combination therapies targeting multiple hallmarks of aging simultaneously. Because aging is driven by interconnected biological processes rather than a single molecular pathway, multidrug approaches may ultimately prove more effective than monotherapy. Rational combinations integrating metabolic modulators, senotherapeutics, mitochondrial-targeted agents, and lifestyle interventions deserve particular attention in future randomized clinical trials.

### 7.1. Combination Therapies

Aging is a multifactorial process involving numerous cellular pathways. It is unlikely that a single drug will effectively influence all aspects of this process. Combination regimens are expected to become an important direction of future geroprotective research—for example metformin (AMPK activator) combined with rapamycin (mTOR inhibitor), senolytics, and NAD^+^ precursors [193].

Preclinical data indicate additive or synergistic effects of such combinations [194].

### 7.2. Development and Validation of Biomarkers

Reliable biomarkers of biological aging are essential for effective translation. Advances in epigenetic clocks, proteomics, metabolomics, and functional frailty markers provide great hope, but regulatory acceptance remains limited [186,194].

Future clinical trials must integrate biomarkers in order to assess efficacy, stratify patients, and shorten study duration.

### 7.3. Personalized Medicine and Pharmacogenomics

The effectiveness of geroprotective drugs may depend on genetic background, metabolic profile, microbiota, and comorbidities. Pharmacogenomics may facilitate identification responders and non-responders to therapies, enabling personalization [195].

For example, polymorphisms in AMPK or mTOR pathways may influence the response to metformin or rapamycin.

### 7.4. Expansion of Clinical Studies

Current human studies are mainly pilot trials of small size and short duration. Future research should prioritize large, multicenter randomized studies evaluating both biomarkers and hard endpoints (multimorbidity, disability, mortality). Innovative designs, including adaptive and pragmatic studies, may accelerate translation [191].

### 7.5. Integration with Lifestyle

Pharmacological geroprotection should not be considered separately from lifestyle [2]. Caloric restriction, physical activity, and diet modulate the same molecular pathways that are targeted by drugs including nutrient-sensing pathways such as AMPK/mTOR signaling, sirtuin-dependent regulation of cellular stress responses, and PGC-1α–driven mitochondrial biogenesis. Integration of pharmacological therapies with behavioral interventions may be the most effective strategy for extending healthy life [1,192,196].

In summary, the future of research on geroprotective pharmacology requires interdisciplinary collaboration, biomarker validation, therapy personalization, and ethical large-scale clinical studies [196]. The ultimate goal is not only lifespan extension, but above all improvement of quality of life and preservation of functional independence in older age. Future randomized trials should incorporate biological age measurements, molecular biomarkers, functional outcomes, and multimorbidity-based endpoints to better evaluate true geroprotective efficacy.

## 8. Translational Perspectives: From Geroscience to Clinical Development

The translational gap in geroprotective pharmacology does not necessarily imply that candidate interventions must be evaluated exclusively in healthy aging populations. An alternative strategy is to investigate geroscience-based interventions in age-related diseases characterized by well-defined clinical endpoints and substantial involvement of fundamental mechanisms of biological aging. Such disease-oriented trials may provide a more feasible pathway for establishing safety, target engagement, and clinically meaningful efficacy while generating evidence relevant to broader geroprotective applications.

Idiopathic pulmonary fibrosis (IPF), for example, represents a clinically relevant setting in which cellular senescence, chronic inflammation, extracellular matrix remodeling, and tissue dysfunction converge. Clinical development of interventions targeting these mechanisms illustrates how disease-focused trials may facilitate the translation of geroscience concepts into clinically actionable therapies [1,186,191,192]. Importantly, evidence obtained in specific age-related diseases should not automatically be extrapolated to generalized geroprotection. Rather, successful disease-oriented translation may represent an intermediate step toward testing whether modulation of shared aging mechanisms can ultimately delay multimorbidity and preserve healthspan across broader populations.

## 9. Conclusions

Pharmacological targeting of conserved aging pathways represents a promising direction in translational geroscience. Current evidence is strongest at the mechanistic and preclinical levels, whereas clinically validated geroprotection in humans remains limited. Metformin and rapamycin remain among the most extensively investigated candidates, while SGLT2 inhibitors, GLP-1 receptor agonists, senolytics, NAD^+^ precursors, and emerging epigenetic approaches represent interventions at different stages of translational development.

The principal uncertainties concern long-term efficacy and safety, appropriate patient selection, treatment timing, and the absence of universally validated biomarkers and clinically accepted aging-related endpoints. Importantly, modulation of aging-associated molecular pathways or biomarkers should not be considered equivalent to demonstrated improvement in human healthspan. The central translational challenge is therefore not the identification of additional pathways or candidate compounds alone, but the demonstration that their modulation produces durable, clinically meaningful benefits in humans.

Future research should prioritize adequately powered randomized trials incorporating standardized biological-age measures together with clinically meaningful outcomes such as multimorbidity, disability-free survival, functional independence, and mortality. Biomarker-guided precision medicine, appropriate treatment timing and duration, and integration of pharmacological interventions with preventive lifestyle strategies may help improve the design and interpretability of future geroscience trials [192,197].

## Figures and Tables

**Table 1:** Pharmacological Candidates with Geroprotective Potential.

Intervention	Main Molecular Target/Mechanism	Preclinical Evidence	Clinical Evidence	Main Limitations
Metformin	AMPK activation, indirect mTOR inhibition, mitochondrial complex I modulation, anti-inflammatory and senomorphic effects	Extended lifespan and delayed age-related phenotypes in animal models	Extensive observational evidence and several randomized trials support metabolic and cardiovascular benefits. However, recent randomized studies (including MET-PREVENT) failed to demonstrate improvement in physical function or frailty-related outcomes, and evidence for direct geroprotection remains inconclusive.	Lack of definitive randomized evidence for lifespan extension in humans
Rapamycin/Rapalogs	mTORC1 inhibition, autophagy activation, modulation of immunosenescence and SASP	Robust lifespan extension demonstrated across multiple species	Small randomized clinical trials demonstrate biological activity and acceptable safety at low intermittent doses, but no convincing evidence currently exists for slowing biological aging or extending lifespan in humans.	Potential immunosuppression, metabolic adverse effects, limited long-term safety data
Statins	HMG-CoA reductase inhibition, anti-inflammatory and endothelial-protective effects	Anti-inflammatory, antioxidant and endothelial protective effects. Robust animal studies have not consistently demonstrated lifespan extension despite favorable effects on aging-related pathways.	Observational studies suggest reduced incidence of several age-related diseases; however, randomized clinical trials evaluating geroprotective effects are lacking.	No convincing evidence of delayed biological aging or lifespan extension in humans.
SGLT2 Inhibitors	Glycosuria induction, AMPK modulation, reduction in inflammation and oxidative stress	Improved median lifespan and reduced inflammaging in animal models	Large randomized studies demonstrate cardioprotective and nephroprotective benefits	Lack of aging-specific clinical trials; risk of ketoacidosis and genitourinary infections
GLP-1 Receptor Agonists	GLP-1 receptor signaling, metabolic regulation, anti-inflammatory and neuroprotective effects	Improved metabolic parameters, cognition, and physical performance in aged animals	Preliminary evidence from post hoc analyses suggests possible effects on epigenetic aging biomarkers; however, confirmation in dedicated randomized geroscience trials is lacking.	No direct evidence for lifespan extension; gastrointestinal adverse effects and high costs
Senolytics	Selective elimination of senescent cells through SCAP inhibition	Improved physical performance, reduced senescent cell burden, and lifespan extension in animal models	Early clinical studies demonstrate biological activity and acceptable safety, but evidence remains insufficient to conclude that senolytics delay biological aging or improve healthspan.	Limited human data; safety, selectivity, and optimal dosing remain uncertain
NAD^+^ Precursors (NR/NMN)	Restoration of intracellular NAD^+^ levels, activation of sirtuins and mitochondrial pathways	Improved mitochondrial function, insulin sensitivity, and metabolic parameters	Increased NAD^+^ levels and selected metabolic improvements observed in small clinical studies	Lack of long-term clinical outcome data and heterogeneous study results
Taurine	Antioxidant activity, mitochondrial support, modulation of inflammation and cellular senescence	Extended lifespan and improved healthspan parameters in animal models	Small studies demonstrated reduction in oxidative stress and cardiometabolic improvements	No evidence for lifespan extension in humans; insufficient long-term RCTs
Menopausal Hormone Therapy (MHT)	Estrogen receptor signaling, modulation of inflammation, mitochondrial function, and vascular aging	Experimental evidence suggests modulation of several hallmarks of aging	Effective in treatment of menopausal symptoms and prevention of osteoporosis; possible favorable effects when initiated early after menopause	Timing-dependent effects; thromboembolic risk; not recommended solely for anti-aging purposes
Epigenetic Modulators/Partial Reprogramming	Modulation of epigenetic drift, histone acetylation, and cellular reprogramming pathways	Reversal of selected aging signatures in vitro and animal models	Very early-stage clinical evaluation	Risk of tumorigenesis; lack of long-term efficacy and safety data

Abbreviations: AMPK, AMP-activated protein kinase; mTOR, mechanistic target of rapamycin; SASP, senescence-associated secretory phenotype; SGLT2, sodium-glucose cotransporter 2; GLP-1, glucagon-like peptide-1; SCAP, senescence-associated anti-apoptotic pathway; NAD^+^, nicotinamide adenine dinucleotide; NR, nicotinamide riboside; NMN, nicotinamide mononucleotide; RCTs, randomized controlled trials; MHT, menopausal hormone therapy.

**Table 2:** Major Ongoing and Recent Clinical Trials Evaluating Geroscience-Based Interventions.

Study/Intervention	Population	Main Endpoint/Objective	Key Translational Finding	Status
TAME—metformin	Adults aged 65–79 years without diabetes	Time to occurrence of a composite of multiple age-related diseases	Designed to test whether targeting aging biology can delay the onset of multimorbidity rather than a single age-related disease	Planned; not yet launched
PEARL—intermittent rapamycin	Healthy adults undergoing normative aging	Safety, visceral adiposity, metabolic and healthspan-related outcomes	Randomized clinical evaluation of intermittent rapamycin; the primary visceral-adiposity endpoint was not significantly changed, although selected secondary outcomes showed potential benefits	Completed; results published
Dasatinib + quercetin (D+Q)	60 postmenopausal women	Bone resorption and bone formation markers	Primary endpoint was not significantly different between groups; transient changes in bone-formation markers and exploratory subgroup responses were observed	Completed; Phase II
SENIOR—D+Q/nicotinamide riboside	Adults aged 60–90 years with osteopenia/osteoporosis and increased fracture risk	Bone turnover markers, including CTX and markers of bone formation	Tests whether senescence- and NAD^+^-targeting interventions can modify age-related bone loss	Phase II; completed; results not yet reported
Senolytic therapy in age-related disease	Older adults with conditions including idiopathic pulmonary fibrosis and chronic kidney disease	Disease-specific clinical, functional and senescence-related outcomes	Provides early clinical testing of whether targeting senescent cells translates into clinically meaningful benefit	Early clinical development
NAD^+^ precursor interventions (NR/NMN)	Older adults/metabolically characterized populations	NAD^+^ availability and metabolic or functional outcomes	Human studies demonstrate biological activity, but clinically meaningful geroprotection remains unestablished	Early clinical development

Abbreviations: TAME, Targeting Aging with Metformin; PEARL, Participatory Evaluation of Aging with Rapamycin for Longevity; D+Q, dasatinib plus quercetin; SENIOR, Senolytics in Older Adults; CTX, C-terminal telopeptide; NAD^+^, nicotinamide adenine dinucleotide; NR, nicotinamide riboside; NMN, nicotinamide mononucleotide.

**Table 3:** Evidence Matrix for Candidate Geroprotective Interventions.

Intervention	Key Preclinical Findings	Human Observational Evidence	Clinical Trial Evidence	Biomarker Evidence	Evidence Strength	Main Limitations
Metformin	Extends lifespan and delays age-related phenotypes in multiple animal models; modulates AMPK, mTOR, mitochondrial ROS, and SASP [35,58]	Lower incidence of cancer, cardiovascular diseases, and reduced all-cause mortality in observational cohorts [19,20,59,60]	No completed large-scale RCT demonstrating lifespan or healthspan extension; TAME trial not yet initiated [21]	Improvements in inflammatory and metabolic markers; possible modulation of senescence-associated pathways [46,54]	Moderate	Predominantly observational human evidence; lack of hard aging endpoints; uncertainty regarding optimal dose and treatment timing
Rapamycin/Rapalogs	Robust lifespan extension across multiple species; improves immune and metabolic aging phenotypes [7,16,32,33,34,35]	Limited observational data in longevity-user cohorts [106]	Small clinical trials suggest improved vaccine response and possible effects on biological aging markers [13,129,132,133]	Reduction in p16^INK4a expression; modulation of DNA damage response and immunosenescence [68,129]	Low-to-Moderate	Small studies; heterogeneous endpoints; lack of long-term randomized clinical data
Senolytics (Dasatinib + Quercetin, Fisetin, Navitoclax)	Reduced senescent cell burden; improved physical function and lifespan in mice [28,29,95,96,97]	Very limited	Early pilot studies in IPF, CKD, and osteoporosis/frailty-related settings [113,114,115,116,117,118,119,140,141]	Reduction in senescence-associated transcripts and SASP markers [113,114]	Low	Small studies; short follow-up; safety concerns; lack of standardized senescence biomarkers
NAD^+^ Precursors (NR/NMN)	Improved mitochondrial function, insulin sensitivity, and metabolic parameters in animal models [41,42]	Limited	Small clinical trials demonstrated increased NAD^+^ levels and selected metabolic improvements [99,100,100,151,152,153]	Increased NAD^+^ concentrations; variable metabolic and muscle-related effects [121,122,123,124]	Low-to-Moderate	Heterogeneous studies; inconsistent clinical effects; absence of hard clinical endpoints
SGLT2 Inhibitors	Improved median lifespan in male mice; reduced inflammation and SASP activity [69,70,71,72,73,74]	Strong cardiovascular and renal outcome data in diabetes populations [71]	Large randomized clinical trials demonstrate cardiovascular and nephroprotective benefits, but not primary geroprotective endpoints [78]	Reduction in inflammatory markers; modulation of AMPK and nutrient-sensing pathways [69,70]	Moderate	No trials in healthy aging populations; uncertain effects on biological aging
GLP-1 Receptor Agonists	Neuroprotective, anti-inflammatory, and metabolic benefits in animal models [76,77,78]	Strong observational and metabolic outcome data	Large randomized clinical trials demonstrate cardiovascular benefits and weight reduction [129,130,131]	Preliminary epigenetic aging modulation reported in selected populations [132]	Low-to-Moderate	Cardiovascular benefits well established, but direct evidence for slowing biological aging or extending lifespan in humans remains limited
Statins	Anti-inflammatory and endothelial-protective effects; inconsistent lifespan effects in animals [64,65]	Reduced cardiovascular events and mortality in high-risk populations [66,67]	Strong cardiovascular RCT evidence, but trials were not designed to evaluate geroprotection	Limited evidence for modulation of aging biomarkers	Low-to-Moderate	Confounding in observational studies; no evidence of lifespan extension in healthy individuals
Menopausal Hormone Therapy (MHT)	Estrogen signaling modulates several hallmarks of aging in experimental studies [42,47]	Associations with lower biological aging discrepancy and reduced mortality in selected cohorts [82,83,84]	Large randomized clinical trials support symptom control and selected cardiometabolic benefits depending on treatment timing [134,135,136]	Possible effects on epigenetic and inflammatory aging markers [84]	Low-to-Moderate	Timing-dependent effects; controversial risk-benefit profile; not recommended for primary anti-aging prevention
Epigenetic Modulators/Partial Reprogramming	Reversal of epigenetic aging signatures in vitro and animal models [94,95,96]	None	Very early-phase human studies only [97,98]	Reversal of epigenetic age in experimental systems	Very Low	Potential tumorigenesis; lack of long-term safety and efficacy data
Natural Polyphenols (Resveratrol, Curcumin, EGCG, Quercetin)	Activation of the AMPK–SIRT1 axis, inhibition of NF-κB signaling, enhanced autophagy, and reduced oxidative stress in cellular and animal models [36,37,38,40,41,47,48,64,65,100]	Limited and heterogeneous	Small heterogeneous clinical trials with inconsistent outcomes	Improvements in oxidative stress and inflammatory markers in selected studies	Low	Poor bioavailability; inconsistent human evidence; lack of standardized formulations
Taurine	Increased lifespan and improved healthspan parameters in mice and primates [91]	Associations with favorable metabolic profiles [91]	Small randomized clinical trials suggest reduced oxidative stress and cardiometabolic improvements [92,93]	Reduction in CRP and oxidative stress markers [91,92,93]	Low-to-Moderate	No evidence of lifespan extension in humans; limited long-term clinical data

Evidence strength represents the authors’ qualitative assessment of the consistency, quality, and translational maturity of evidence specifically supporting a geroprotective effect, considering preclinical findings, human observational and biomarker studies, and randomized clinical trials. Greater weight was given to reproducible human clinical outcomes and validated aging-related endpoints. The classification is intended for comparative purposes and does not represent a formal evidence-grading system such as GRADE. Abbreviations: AMPK, AMP-activated protein kinase; mTOR, mechanistic target of rapamycin; ROS, reactive oxygen species; SASP, senescence-associated secretory phenotype; RCT, randomized controlled trial; TAME, Targeting Aging with Metformin; p16^INK4a, cyclin-dependent kinase inhibitor 2A (CDKN2A); IPF, idiopathic pulmonary fibrosis; CKD, chronic kidney disease; NAD^+^, nicotinamide adenine dinucleotide; NR, nicotinamide riboside; NMN, nicotinamide mononucleotide; SGLT2, sodium-glucose cotransporter 2; GLP-1, glucagon-like peptide-1; MHT, menopausal hormone therapy; SIRT1, sirtuin 1; NF-κB, nuclear factor kappa B; EGCG, epigallocatechin gallate; CRP, C-reactive protein.

## Data Availability

No new data were created or analyzed in this study. Data sharing is not applicable to this article.

## Abbreviations

The following abbreviations are used in this manuscript:

AMPK	AMP-activated protein kinase
CKD	chronic kidney disease
CRP	C-reactive protein
CV	cardiovascular
EGCG	epigallocatechin gallate
GLP-1	glucagon-like peptide-1
GLP-1RA	GLP-1 receptor agonist
GIP	glucose-dependent insulinotropic peptide
GI	gastrointestinal
HMG-CoA	3-hydroxy-3-methylglutaryl-coenzyme A
IL-6	interleukin 6
IPF	idiopathic pulmonary fibrosis
mTOR	mechanistic target of rapamycin
mTORC1	mechanistic target of rapamycin complex 1
NAD^+^	nicotinamide adenine dinucleotide
NMN	nicotinamide mononucleotide
NR	nicotinamide riboside
RCT	randomized controlled trial
SASP	senescence-associated secretory phenotype
SCAPs	senescent cell anti-apoptotic pathways
SGLT2i	sodium–glucose cotransporter type 2 inhibitor
SnCs	senescent cells
T2DM	type 2 diabetes mellitus
TAME	targeting aging with metformin
TNF-α	tumor necrosis factor-α
